# Understanding public perceptions toward sustainable healthcare through psychological network analysis of material preference and attitudes toward plastic medical devices

**DOI:** 10.1038/s41598-023-45172-6

**Published:** 2023-10-20

**Authors:** Monique Chambon, Janneke E. Elberse, Jonas Dalege, Nick R. M. Beijer, Frenk van Harreveld

**Affiliations:** 1 National Institute for Public Health and the Environment (RIVM), Bilthoven, The Netherlands; 2 University of Amsterdam, Amsterdam, The Netherlands; 3 Santa Fe Institute, Santa Fe, USA

**Keywords:** Health policy, Psychology and behaviour

## Abstract

Recent and potential future health-care users (i.e., the public) are important stakeholders in the transition toward environmentally sustainable healthcare. However, it remains unclear whether, according to the public, there is room for sustainable innovations in materials for plastic medical devices (PMD). This study explores preferences regarding conventional or bio-based PMD, and psychological mechanisms underlying these preferences. We administered two surveys among Dutch adults from a research panel. Results from the first survey (i.e., open-text survey on attitude elements; *N*^Study1^ = 66) served as input for the second survey (i.e., Likert-scale survey on beliefs, emotions, perceived control, social norms, trust, related to current and bio-based PMD, and health and age; *N*^Study2^ = 1001; *M*^*age*^ = 47.35; 54.4% female). The second survey was completed by 501 participants who, in the last two years, received care in which PMD were used, and 500 participants who did not. Cross-sectional psychological networks were estimated with data from the second study using the EBICglasso method. Results showed that participants preferred bio-based over conventional PMD, and this applied regardless of whether devices are used inside or outside of the body. Results also showed emotions play an important role, with emotions regarding bio-based PMD being strongly related to preference. Furthermore, comparing recent and potential future receivers of PMD revealed differences in preference but comparable relations between preference and other psychological variables. This study shows that receivers’ perspectives should not be seen as potential barriers, but as additional motivation for transitioning toward sustainable healthcare. Recommendations for implementation are discussed.

## Introduction

The transition toward environmentally sustainable healthcare is important because of this sector’s relatively high contribution to climate change. For example, the Dutch health-care sector is responsible for 7.3% of the national climate change footprint^1^. However, it is also a particularly challenging sector for sustainable innovations due to strict regulations aimed at protecting patient health and safety. Such regulations may be perceived to impede circular strategies such as the reuse or recycling of products or materials. Nevertheless, several initiatives underline the importance of, and the sector’s commitment to, sustainable healthcare. Examples from the Netherlands are the ‘Green Deal’ between the government and other partners to implement sustainability plans^2^, and a proposal from a governmental advisory body to incorporate sustainability in legislation on values of good patient care^3^. Both initiatives also relate to the sustainability of medical devices. The current study focuses on Plastic Medical Devices (PMD), such as tubes, syringes, luer connectors and bags for blood and medicine to personal protection equipment for staff and medical packaging. Their production often requires non-renewable materials, which contributes to the depletion of natural resources. Many PMD are intended for single use, and this short life cycle contributes to the sector’s plastic waste. Given that medical devices are already the subject of ongoing innovations, for instance with improvements in functionalities or ease of use, it is important to examine whether there is also room to make the process more sustainable. Fortunately, despite challenges from a regulatory perspective, an increasing amount of research is conducted on improving the sustainability of medical devices^4,5^.

Innovation processes of medical devices are mostly guided by legislation and policy, without including perspectives of people who receive health care or potential future care users (i.e., the public)^6^. However, generally speaking, innovations involve not only opportunities but also unknowns. In a medical context such unknowns could impose potential health risks, therefore it is important to include people’s perceptions of such innovations. In addition to innovations affecting the public directly as (future) health-care users, it impacts them indirectly because sustainable innovations mitigate climate change and thus the adverse effects of health care on the public’s health and living environment. Moreover, public perceptions could impact the transition toward sustainable healthcare and, more specifically, to more sustainable medical devices. That is, positive attitudes toward sustainable alternatives could foster or even accelerate the transition, whereas negative attitudes could potentially obstruct it. The latter could happen if societal unrest or fear related to medical devices results in people avoiding health care. As such, the transition toward sustainable healthcare can be considered a societal challenge, and research into stakeholder’s perspectives on sustainable innovations should include both patients and other people from the public. However, previous research mainly includes health-care *providers* as stakeholders, and health-care *users* (i.e., patients) less so^7,8^. Notable exceptions are found in research on reprocessing and reusing single-use medical devices^9,10^.

Accordingly, the aim of this study is to investigate whether there is room for sustainable innovations in PMD according to the public and does so by including perspectives of both health-care users that were exposed to PMD in the last two years (from here on recent PMD receivers) and others (from here on potential future PMD receivers). More specifically, we examine preferences for conventional PMD or bio-based PMD (i.e., PMD made from biomass). Bio-based PMD was chosen as an alternative to conventional PMD based on two considerations. First, the alternative had to be perceived as more sustainable than conventional PMD by participants. Second, it had to be an alternative to which patients are directly exposed themselves so that the alternative would alter people’s exposure to PMD. This in contrast to an alternative that focuses on the end of the life cycle of PMD such as materials optimized for only recycling. The choice for bio-based PMD was based on a review study that concludes that bio-based PMD could potentially reduce the environmental impact of PMD significantly compared to conventional PMD^11^. Whether bio-based PMD or conventional PMD have the lowest environmental impact could only be assessed specifically case by case, however, participants were expected to perceive a bio-based alternative as the more sustainable option. Additionally, we explore how psychological variables related to health, safety, and sustainability are related to this preference. Insight into such relations can provide an important step toward identifying relevant psychological elements for stimulating acceptance of sustainable PMD innovations. This study approaches preference as a behavioral intention (i.e., hypothetical choice between conventional or bio-based PMD), therefore the psychological variables included in this study cover the elements preceding behavioral intentions in the Theory of Planned Behavior^12^, that is, attitudes (i.e., beliefs and emotions), social norms and perceived control. Other variables that were included because of their expected relevance for preference are ambivalence, based on previous research into bio-based plastic in a consumer context^13,14^; and trust, because of its expected relevance for risk perception^15^. Moreover, the variables are included for both conventional and bio-based PMD, to explore whether these differ in their relation to preference.

The current study adopts a network methodology to shed light on the interplay of such a diverse set of psychological variables. This explorative, data-driven approach is based on the Causal Attitude Network (CAN) model^16^, which conceptualizes attitudes as a complex system of cognitive, affective and behavioral elements that form attitudes. In such networks, attitude elements are displayed as nodes, and (linear) relations between them that are estimated with survey data are displayed as edges. This approach has already been applied in the context of attitudes toward bio-based plastic in a consumer context^13^. We extend the CAN model beyond attitudes with the aforementioned psychological variables, similar to previous research into broad attitude networks in the context of health behavior^17–20^. This approach allows us to describe the complex interplay of these variables in visually insightful networks, as well as compare groups to examine whether differences can be observed between the networks of recent and potential future PMD receivers. This could inform us about the approach to stakeholder involvement: meaningful differences between these groups suggest that they should be involved separately, which might make it more challenging, whereas the absence of meaningful differences suggests that involving the general public can provide insights relevant for both groups.

## Method

This research was approved by the Ethical Review Board of the University of Amsterdam (022-SP-14541). All research was performed in accordance with relevant guidelines and regulations. Informed consent was obtained from all participants. In total, two studies were conducted. It applies to both studies that participants were recruited via a research panel (Ipsos), the original survey was presented in Dutch, and the survey was programmed in Qualtrics.

### Study 1

The first study aimed to identify relevant attitude elements that served as input for the items tapping into attitudes in the survey administered in Study 2. To do so, health-care users that were exposed to PMD in the last two years (*N*^*Study 1*^ = 66) were asked to list their beliefs (i.e., advantages and disadvantages) and emotions regarding the use of PMD. The first section provided only general instructions; the second section repeated the questions with the instruction to keep in mind the effects of the use of PMD for health, safety, and the environment. More information on the method and results of Study 1 can be found in Appendix 1.1. In summary, the following themes were identified in the open-text responses and used as input for the survey. Respondents reported beliefs about the use of PMD related to hygiene, ease of use and availability for health-care professionals, their necessity, durability, quality, material properties, costs and their necessity of use, but also their environmental pollution, waste volume and management, recyclability, long- and short-term health consequences, unnecessarily usage and raw materials for production. Regarding emotions, respondents reported both positive and negative feelings, and mentioned safety, guilt, and concerns about both safety and environmental consequences. Finally, their answers referred to the presence or absence of awareness about the use of PMD. Participants’ terminology also served as input for formulating the corresponding survey items in Study 2.

### Study 2

#### Participants

In total, 1001 participants completed the survey (*N*^*Study 2*^ = 1001). This total sample contained both people who reported that, in the last two years, they received health care in which PMD were used (*n* = 501; from here on called the high relevance subsample) and people who reported that, in the last two years, they received either no health care or health care in which no PMD were used (*n* = 500; from here on called the low relevance subsample; see Appendix 1.2 for the items for subsample allocation). Around 500 respondents per subsample was expected to provide sufficient power to accurately estimate networks: a moderately sized network (maximum of 30 variables) calculated with continuous data from a sample of 250 respondents is likely to result in accurate network estimation^21^. Additionally, network stability checks were conducted after data collection. Only participants who passed at least one of two attention checks were allowed to complete the survey.

Table 1 provides the sample’s demographic information. The total sample was broadly comparable to the Dutch adult population in terms of age and gender (slightly more females). This cannot be specified for the relevance subsamples, since it is unknown how the population is distributed over these subsamples.

**Table 1:** Demographic information of the total sample and the relevance subsamples.

Sample	Total^a^ (*N* = 1001)		Low relevance (*n* = 500)		High relevance (*n* = 501)	
*Age*	*M (SD)*	*Mdn*	*M (SD)*	*Mdn*	*M (SD)*	*Mdn*
Age (years)^b^	47.35 (15.55)	48	46.5 (15.23)	47	48.21 (15.84)	49
*Gender*	*n*	%	*n*	%	*n*	%
Male	453	45.3%	239	47.8%	214	42.7%
Female	545	54.4%	261	52.2%	284	56.7%
Other	3	0.3%	0	0.0%	3	0.6%
*Education*	*n*	%	*n*	%	*n*	%
Primary	210	21.0%	112	22.4%	98	19.6%
Secondary	451	45.1%	223	44.6%	228	45.5%
Higher	340	34.0%	165	33.0%	175	34.9%

^a^The monthly screener questionnaire that Ipsos sends to their entire research panel showed that approximately 26% of the panel qualifies as high relevance and 67% as low relevance (7% did not want to answer these questions).

^b^Age of the participants ranged from 18 to 90 years.

#### Measures and procedure

**Figure 1:**
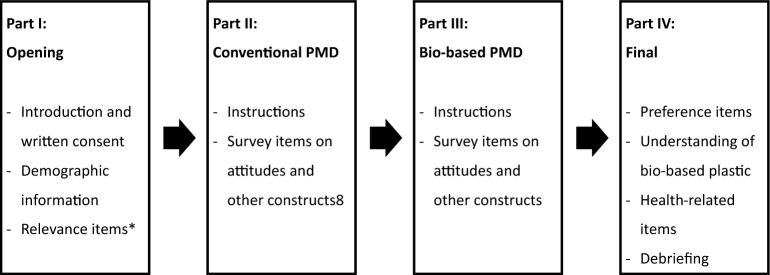
Survey flow and elements. *Answers determined allocation to relevance subsamples (or exclusion from survey; see appendix 1.2 for more information). The rest of the survey was identical for both subsamples.

All survey items are provided in Appendix 1.2 and Fig. 1 presents the survey’s flow and its elements. After a short introduction and obtaining written consent, the survey started with questions about demographics and relevance. After presenting a definition of PMD, the items on conventional PMD were administered. Subsequently, a definition of bio-based plastic was provided (i.e., made from biomass), followed by the statement that the difference between bio-based and regular PMD lies in its materials, after which the items on bio-based PMD were administered. Note that no statements on environmental impact or sustainability were included. Such a brief introduction is expected to approach a rather realistic setting, assuming that the implementation of PMD from alternative materials is accompanied by little or no information toward patients. Participants were instructed to answer questions about bio-based PMD as if they were already being used in healthcare. The items in the bio-based section were phrased like the items on conventional PMD but with the word ‘bio-based’ inserted before PMD. Also, the bio-based items included the phrasing ‘…, compared to current plastic medical devices, …’. Items from the section on conventional PMD that could not be answered from a hypothetical situation were not included in bio-based items (e.g., ‘Plastic medical devices are cheap’). Within the sections on conventional and bio-based PMD, items were presented in clusters (i.e., general attitude, beliefs, emotions, control and social norms, trust), and within these clusters the order of items were randomized where possible. The last part of the survey contained items on material preference for PMD that a) do *not* come into contact with the body, b) come into contact with the *outside* of the body, and c) come into contact with the *inside* of the body. This distinction was inspired by European legislation on categories of medical devices and corresponding regulations based on their risk for patients. Participants indicated what material they would choose, ranging from 1 (definitely current plastic) to 7 (definitely bio-based plastic). Finally, participants answered a question about their understanding of bio-based plastic and then completed health-related items.

After collecting the data, survey items were combined into variables to include in the network (see Appendix 1.3 for a detailed overview). Table 2 provides an overview of these variables, including their interpretation and example survey items. Variables can consist of either a single item or the mean score on multiple items (except for the ambivalence variable that was calculated with a formula; see Appendix 1.3). In case a variable was calculated with multiple items, the set of items was either predetermined based on theoretical constructs or based on results of a dimension reduction analysis. The latter was conducted for the items covering beliefs and emotions.

**Table 2:** The psychological variables included in this study including the number of items per variable, its interpretation and example items from the survey. Labels of nodes related to bio-based plastic medical devices start with Bio_; labels of nodes related to current plastic medical devices start with Cur_. See Appendix 1.2 for a complete overview of the items included in the survey and Appendix 1.3 for a complete overview of the items per combined variable.

Variable label (no. of items; *a* total sample)	Example items from survey	Interpretation higher score (PMD = plastic medical devices)
Age (1)	What is your age?	Older participant (scale: years)
Bio_Ambivalence (2—formula)	When you only think about the positive [/negative] aspects and ignore the negative [/positive] aspects, how positive [/negative] are you about the use of *bio-based *plastic medical devices in healthcare?	More ambivalence toward bio-based PMD (scale range from 1 to 10)
Bio_BeliefsEnvironment (5; *a* = .80)	The use of *bio-based* plastic medical devices is, compared to regular plastic medical devices,… Much worse for the environment (1)—No difference (4)—Much better for the environment (7)^a^/The use of *bio-based* plastic medical devices produces, compared to regular plastic medical devices,… Waste that is more difficult to degrade (1)—No difference (4)—Waste that is more easily degradable (7)^a^	Beliefs that bio-based PMD are better for the environment than current PMD
Bio_BeliefHealthLongterm (1)	The use of *bio-based* plastic medical devices is, compared to regular plastic medical devices,… Much worse for long-term health (1)—No difference (4)—Much better for long-term health (7)^a^	Belief that bio-based PMD are better for one’s health in the long term than current PMD
Bio_BeliefsSafety (3; *a* = .77)	The use of *bio-based* plastic medical devices is, compared to regular plastic medical devices,… Much less safe for patients (1)—No difference (4)—Much safer for patients (7)^a^/*Bio-based* plastic medical devices are, compared to regular plastic medical devices,… Of much worse quality (1)—No difference (4)—Of much better quality (7)^a^	Beliefs bio-based PMD are safer than current PMD
Bio_BeliefUse (1)	*Bio-based* plastic medical devices… Are for single use (1)—Can be used very often (7)	Belief that bio-based PMD can be reused
Bio_PerceivedControl (1)	I think I have influence over the use of *bio-based* plastic medical devices	Stronger perceived control over the use of bio-based PMD
Bio_Emotions (6; *a* = .84)	About the use of *bio-based* plastic medical devices, compared to regular plastic medical devices, I feel,… Much less guilty (1)—No difference (4)—Much more guilty (7)^a^/About the use of *bio-based* plastic medical devices, compared to regular plastic medical devices, I feel,… Much less concerned about the environment (1)—No difference (4)—Much more concerned about the environment (7)^a^	More positive emotions related to bio-based PMD than current PMD
Bio_Norm (1)	I think other people favor the use of *bio-based* plastic medical devices in healthcare	Stronger social norm favoring bio-based PMD
Bio_Trust (3; *a* = .86)	I trust manufacturers of *bio-based* plastic medical devices. /I trust laws and regulations about *bio-based* plastic medical devices	More trust in bio-based PMD
Cur_Ambivalence (2–formula)	When you only think about the positive [/negative] aspects and ignore the negative [/positive] aspects, how positive [/negative] are you about the use of plastic medical devices in healthcare?	More ambivalence toward current PMD (scale range from 1 to 10)
Cur_BeliefsNegative (6; *a* = .82)	The use of plastic medical devices is a problem due to the raw materials used in production. /The use of plastic medical devices is bad for the environment. /The use of plastic medical devices is bad for patients' long-term health. For example, due to harmful substances or microplastics	More negative beliefs about current PMD
Cur_BeliefsPositive (8; *a* = .81)	Plastic medical devices are easy to use for health-care workers. /Plastic medical devices have important properties. For example, light or flexible. /The use of plastic medical devices is hygienic	More positive beliefs about current PMD
Cur_BeliefRecycle (1)	Plastic medical devices are… Not at all recyclable (1)—Highly recyclable (7)	Belief that current PMD are recyclable
Cur_BeliefUse (1)	Plastic medical devices are… For single use (1)—Highly reusable (7)	Belief that current PMD can be reused
Cur_ PerceivedControl (1)	I think I have influence over the use of plastic medical devices	Stronger perceived control over the use of current PMD
Cur_EmotionsNegative (5; *a* = .78)	When I think about the use of plastic medical devices, I have negative feelings. For example sad or angry. /When I think about the use of plastic medical devices, I feel guilty	More negative emotions related to current PMD
Cur_EmotionsPositive (1)	When I think about the use of plastic medical devices, I have positive feelings. For example happy or satisfied	More positive emotions related to current PMD
Cur_Norm (1)	I think other people favor the use of plastic medical devices in healthcare	Stronger social norm favoring current PMD
Cur_Thinking (1)	To what extent is the use of plastic medical devices in healthcare a topic that you think about?	Thinking about current PMD more often
Cur_Trust (3; *a* = .82)	I trust manufacturers of plastic medical devices. /I trust laws and regulations about plastic medical devices	More trust toward current PMD
Exposure (1)	In the past two years, how often have you come into contact with plastic medical devices? Scale: Rarely or never (1); Annually (2); Every six months (3); Monthly (4); Weekly (5); Several times a week (6); Daily (7)	Exposed more often to PMD
Health (1)	How is your health in general?	
PreferenceExternal (2; *a* = .87)	Plastic medical devices that do *not* come into contact with the body. For example, bags in which urine is collected or packing material. /Plastic medical devices that come into contact with the *outside* of the body. For example stoma bags or gloves Scale: Definitely current plastic (1)—No preference (4)—Definitely bio-based plastic (7)	Preference for bio-based PMD over current PMD
PreferenceInternal (1)	Plastic medical devices that come in contact with the *inside* of the body. For example, a catheter or tube feeding set Scale: Definitely current plastic (1)—No preference (4)—Definitely bio-based plastic (7)	Preference for bio-based PMD over current PMD

All items were measured on a 7-point Likert scale except for Age (open numeric answer field). Mean scores were calculated for nodes that consisted of more than one item. The only exception is Ambivalence (Bio_Amb and Cur_Ambi): this score was calculated with a formula (see Appendix 1.3).

^a^Note that scale for respondents ranged from -3 to 0 to + 3, respectively.

#### Analysis

##### Preliminary analyses

Dimension reduction analyses to combine items into variables were conducted with Principal Axis Factoring with oblimin rotation due to the expected intercorrelation between items. This was done with the dataset from the total sample. Independent sample t-tests were used to compare variable scores between the relevance subsamples.

##### Network analysis

Networks were estimated with the survey data by using the EBICglasso method for continuous and ordinal variables^21^. This method entails Gaussian Markov random field estimation using graphical LASSO and extended Bayesian information criterion for the selection of optimal regularization parameter^22^. Edges can be interpreted as partial correlations, thus associations between variables after controlling for the effects of the other variables in the network. Analyses were conducted in R^23^, for which we used the packages bootnet^22^ for network estimation, stability and accuracy measures, and difference tests; igraph^24^ for community detection; qgraph^25^ for the centrality plots; and the NetworkComparisonTest^26^ to compare the networks of the subsamples.

## Results

This section will first present descriptive results regarding material preference for PMD and other variables in the survey, after which results from the psychological network analyses will be presented.

### Preference and preliminary analyses

For both PreferenceExternal and PreferenceInternal, most participants reported either a preference for bio-based PMD (i.e., score > 4) or no preference between bio-based and current PMD (i.e., score = 4). 63.8% of participants preferred bio-based plastic over current plastic for medical devices used only outside the body (27.7% no preference; 8.5% preference for current plastic). For medical devices used inside of the body, a total of 45.9% preferred bio-based over current plastic (37.4% no preference; 16.8% preference for current plastic).

Table 3 shows descriptive information of all variables for the total sample and the relevance subsamples, including results of comparative tests between subsamples. On average, participants expressed a preference for bio-based PMD compared to current PMD. This applied to both PMD used inside and outside of the body, although more so for the latter (*p* < 0.001). Although this preference for bio-based materials applied to both relevance subsamples, the high relevance subsample expressed a significantly stronger preference for bio-based PMD used outside of the body (*p* = 0.007). For PMD used inside the body, the subsamples’ preference for bio-based plastic did not differ (*p* = 0.966).

**Table 3:** Descriptive information of all variables for the total sample and relevance subsamples, including results of comparative analyses.

Sample	Total (*N* = 1001)			Low (*n* = 500)			High (*n* = 501)			Comparative analyses: Low versus high relevance				
Variable	*M*	*SD*	*Mdn*	*M*	*SD*	*Mdn*	*M*	*SD*	*Mdn*	*t*	*df*	*p*	*d*	*95% CI*
Age	47.35	15.55	48	46.5	15.23	47	48.21	15.84	49	− 1.74	997.63	.082	− 0.11	[− 0.23, 0.01]
**Bio_Ambivalence**	**4.4**	**2.48**	**4.5**	**4.64**	**2.41**	**5**	**4.17**	**2.54**	**4.5**	**2.96**	**996.36**	.003*	**0.19**	**[0.06, 0.31]**
**Bio_BeliefsEnviron**	**5.31**	**1.02**	**5.4**	**5.19**	**1.05**	**5.2**	**5.43**	**0.97**	**5.4**	**− 3.84**	**992.52**	< .001*	**− 0.24**	**[− 0.37, − 0.12]**
**Bio_BeliefHealthLong**	**4.65**	**1.18**	**4**	**4.51**	**1.11**	**4**	**4.78**	**1.23**	**4**	**− 3.67**	**989.33**	< .001*	**− 0.23**	**[− 0.36, − 0.11]**
Bio_BeliefsSafety	4.13	0.79	4	4.1	0.73	4	4.16	0.85	4	− 1.24	975.44	.215	− 0.08	[− 0.20, 0.05]
Bio_BeliefUse	2.82	1.76	3	2.9	1.71	3	2.75	1.8	2	1.35	996.27	.178	0.09	[− 0.04, 0.21]
Bio_PerceivedControl	3.1	1.57	4	3.11	1.48	4	3.1	1.66	4	0.08	986.09	.934	0.01	[− 0.12, 0.13]
Bio_Emotions	4.72	0.94	4.5	4.69	0.9	4.5	4.75	0.98	4.67	− 1.10	991.33	.272	− 0.07	[− 0.19, 0.05]
Bio_Norm	5.01	1.3	5	4.92	1.22	5	5.09	1.37	5	− 2.08	986.46	.038	− 0.13	[− 0.26, − 0.01]
Bio_Trust	4.7	1.19	4.67	4.65	1.13	4.67	4.75	1.26	4.67	− 1.31	988.10	.189	− 0.08	[− 0.21, 0.04]
Cur_Ambivalence	5.16	2.25	5.5	5.2	2.21	5.5	5.12	2.29	5.5	0.56	997.98	.572	0.04	[− 0.09, 0.16]
Cur_BeliefsNegative	4.76	1.07	4.83	4.69	1.05	4.83	4.84	1.08	4.83	− 2.17	998.29	.030	− 0.14	[− 0.26, − 0.01]
**Cur_BeliefsPositive**	**5.31**	**0.82**	**5.38**	**5.14**	**0.84**	**5.12**	**5.48**	**0.76**	**5.5**	**− 6.78**	**989.48**	< .001*	**− 0.43**	**[− 0.55, − 0.30]**
Cur_BeliefRecycle	4.27	1.71	4	4.31	1.66	4	4.23	1.75	4	0.71	996.23	.479	0.04	[− 0.08, 0.17]
**Cur_BeliefUse**	**2.69**	**1.76**	**2**	**2.9**	**1.8**	**3**	**2.48**	**1.7**	**2**	**3.79**	**995.66**	< .001*	**0.24**	**[0.12, 0.36]**
Cur_PerceivedControl	2.66	1.59	2	2.74	1.5	3	2.59	1.67	2	1.47	988.56	.143	0.09	[− 0.03, 0.22]
Cur_EmotionsNegative	3.27	1.17	3.4	3.35	1.12	3.4	3.19	1.22	3.2	2.18	992.59	.029	0.14	[0.01, 0.26]
**Cur_EmotionsPositive**	**4.27**	**1.47**	**4**	**4.12**	**1.39**	**4**	**4.41**	**1.54**	**4**	**− 3.10**	**989.50**	.002*	**− 0.20**	**[− 0.32, − 0.07]**
Cur_Norm	4.63	1.25	4	4.56	1.18	4	4.7	1.32	4	− 1.78	986.28	.076	− 0.11	[− 0.24, 0.01]
**Cur_Thinking**	**2.72**	**1.74**	**2**	**2.44**	**1.57**	**2**	**2.99**	**1.85**	**3**	**− 5.14**	**974.30**	< .001*	**− 0.32**	**[− 0.45, − 0.20]**
Cur_Trust	4.65	1.25	4.67	4.59	1.18	4.67	4.7	1.32	4.67	− 1.37	986.27	.169	− 0.09	[− 0.21, 0.04]
**Exposure**	**2.31**	**1.64**	**2**	**1.65**	**1.46**	**1**	**2.97**	**1.55**	**3**	**−** **13.81**	**996.00**	< .001*	**−** **0.87**	**[−** **1.00, −** **0.74]**
**Health**	**5.17**	**1.37**	**6**	**5.62**	**1.13**	**6**	**4.72**	**1.44**	**5**	**10.92**	**947.39**	< .001*	**0.69**	**[0.56, 0.82]**
**PreferenceExternal**	**5.25**	**1.54**	**5**	**5.12**	**1.53**	**5**	**5.38**	**1.54**	**5.5**	**− 2.70**	**998.98**	.007*	**− 0.17**	**[− 0.29, − 0.05]**
PreferenceInternal	4.67	1.75	4	4.66	1.65	4	4.67	1.84	4	− 0.04	987.94	.966	− 0.00	[− 0.13, 0.12]

Answers were measured on 7-point Likert scales, except Age (years) and Bio_Ambivalence/Cur_Ambivalence (scale 1–10). Variables that differed significantly between the relevance subsamples are displayed in bold text.

*p < .01, indicating a significant difference between the relevance subsamples (more conservative than *p* < .05 to correct for multiple testing).

### Network analyses total sample

**Figure 2:**
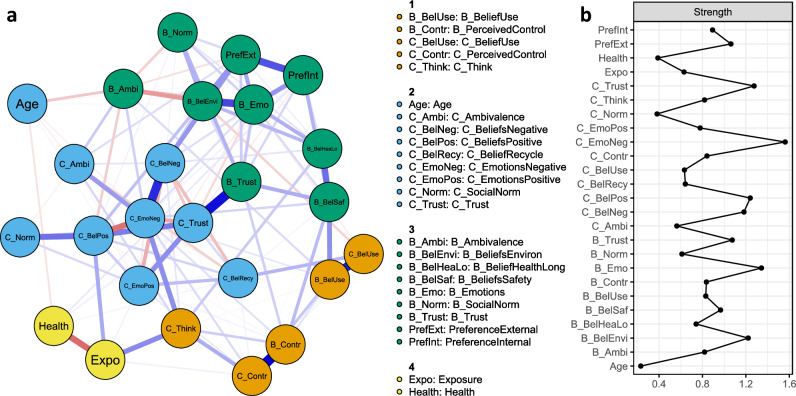
(**a**) Network of psychological variables related to plastic medical devices for the total sample. Nodes represent variables (C_ = related to current PMD, B_ = related to bio-based PMD) and edges represent relations between them (i.e., partial correlations), with blue edges for positive and red edges for negative relations. A positive (negative) relation indicates that people who reported, on average, a higher score on one variable also reported, on average, a higher (lower) score on the other variable, and vice versa. Strength of the relations is indicated by edge width and color density (see Table 4 for corresponding edge weights); (**b**) Node statistic Strength, which represents how connected a node is to the rest of the network (calculated as the sum of a node’s absolute edge weights).

Figure 2a shows the psychological network of variables related to PMD (see Table 4 for corresponding edge weights; edges discussed in text are provided in parenthesis).

**Table 4:** Edge weights of edges in psychological network related to plastic medical devices for the total sample. See Fig. 2 for legend.

	Age	Bio_Ambi	Bio_BelEnvir	Bio_BelHealLong	Bio_BelSafe	Bio_BelUse	Bio_Control	Bio_Emo	Bio_Norm	Bio_Trust	Cur_Ambi	Cur_BelNeg	Cur_BelPos	Cur_BelRecy	Cur_BelUse	Cur_Control	Cur_EmoNeg	Cur_EmoPos	Cur_Norm	Cur_Think	Cur_Trust	Exposure	Health	PrefExt	PrefInt
Age		− .09						.01			− .03						− .03	.02					− .05		.01
Bio_Ambi			− .17			.06		− .11	− .08		.09		− .08			.01	.08							− .05	
Bio_BelEnvir				.12				.31	.13	.06		.08	.10		− .07	.00								.17	
Bio_BelHealLong					.24			.13	.04			.05								.09				.03	.05
Bio_BelSafe						.19	.11	.06		.14				.02		.01	.07			.00					.13
Bio_BelUse							.05						− .01		.37	.09	.04			.01				− .01	
Bio_Control										.08			− .02			.42	.05	.02		.08					
Bio_Emo									.07	.09		.05			− .03		.06	− .03			− .06			.15	.18
Bio_Norm										.13		.02	.02	.00										.05	.06
Bio_Trust																					.40		.02	.08	.07
Cur_Ambi												.04		− .08			.17	− .02	− .02	.07				.06	
Cur_BelNeg													.01	− .11			.36	− .13		.04	− .09			.17	.02
Cur_BelPos														.08	.00		− .23	.09	.24		.22	.14		.00	
Cur_BelRecy															.12	.02	− .02	.11			.07		.02		
Cur_BelUse																.02		.01							
Cur_Control																	.06	.04		.14		.03			
Cur_EmoNeg																		− .04		.19	− .14				.02
Cur_EmoPos																			.07	.00	.19	.02			
Cur_Norm																					.06				
Cur_Think																						.19			
Cur_Trust																							.02		− .03
Exposure																							− .25		
Health																									.04
PrefExt																									.29
PrefInt																									

#### Communities

Communities represents interrelatedness among nodes through clusters of nodes that are more connected to each other than to nodes outside the community. Preference for the type of plastic used for PMD, both for PMD used outside (PreferenceExternal) and inside (PreferenceInternal) the body, were predominantly interrelated with bio-based variables (see community 3; variable groups with similar color).

#### Edges

The variables PreferenceExternal and PreferenceInternal were relatively strongly related (0.29). After controlling for effects of other variables included in the network, PreferenceExternal had relatively strong edges with Bio_BeliefsEnvironment (0.17), Current_BeliefsNegative (0.17), and Bio_Emotions (0.15). These edges were significantly stronger than other edges with PreferenceExternal, except the edge with Bio_Emotions did not differ significantly from that with Bio_Trust. This indicates that a preference for bio-based PMD used outside the body was related to beliefs that bio-based PMD are better for the environment than current PMD, more positive emotions regarding bio-based PMD, and more negative emotions regarding current PMD. PreferenceInternal had relatively strong relations with Bio_Emotions (0.18) and Bio_BeliefsSafety (0.13), indicating a preference for bio-based plastic for PMD used inside the body was related to more positive emotions regarding bio-based PMD and stronger beliefs that bio-based PMD are safe for patients. These edges did not differ significantly, thus were of comparable strength, but the edge between PreferenceInternal and Bio_Emotions was significantly stronger than other edges with PreferenceInternal. The relation between PreferenceInternal and Bio_BeliefsSafety did not differ significantly from Bio_Trust. Corresponding results regarding edge accuracy and difference tests are provided in Appendix 1.4. Generally speaking, edge weights appear stable (reliable) because confidence intervals were not wide.

Sensitivity analyses in which all preference items were combined into one variable revealed that preference had the significantly strongest relation with Bio_Emotions. Sensitivity analyses in which all preference items were included as separate items (i.e., no contact, contact only with the outside, and contact with the inside of the body) showed that Bio_Emotions was linked to all preference items, but mostly to preference regarding PMD that come into contact with the body (either outside or inside). Current_BeliefsNegative was also linked to all preference items, but mostly to preference regarding PMD that have no contact with the body. Bio_BeliefsEnvironment was only associated with preference regarding PMD that have no contact with the body or only the outside. Bio_BeliefsSafety was only linked to preference regarding PMD used inside the body.

#### Node strength

Figure 2b shows the metric node strength (see legend for interpretation). Current_EmotionsNegative had the significantly highest node score, indicating that this variable had the relatively most and/or strongest edges with other nodes in the network. Corresponding values, stability and difference tests are provided in Appendix 1.5.

### Network analyses comparing subsamples

Results from the Network Comparison Test (NCT) showed a non-significant trend toward higher network connectivity for the high relevance subsample. That is, the global strength of the networks (calculated as the total sum of absolute edge weights in the network) was higher for the high relevance subsample (10.21) than the low relevance subsample (8.43), although this difference was not significant (*p* = 0.052). The significant omnibus test (*p* < 0.01) indicated there were significant differences between the relevance subsamples in specific edges between variables, however, none of these were directly relevant for preference. The only significant difference in edges related to either of the preference variables was small (edge weight difference = 0.03) and did not remain after adjusting the *p*-value to < 0.01 to correct for multiple testing. A significant difference between subsamples that was indirectly relevant for preference is the edge between Bio_BeliefsSafety and Bio_Trust (low relevance 0.00; high relevance 0.24, *p* < 0.001). This indicates that the relation between thinking bio-based PMD are safer than current PMD and trust in bio-based PMD was relatively strong in the high relevance subsample and absent in the low relevance subsample. Regarding node strength, Current_EmotionsNegative showed significantly higher node strength in the high relevance subsample (1.62) compared to the low relevance subsample (1.29, *p* = 0.015), but again this did not remain after correcting for multiple testing. Appendix 1.6 provides a complete overview of the network analysis results per subsample and the NCT results.

### Understanding of bio-based plastic

The end of the survey contained an item on the characteristics that participants thought of when thinking about bio-based PMD (multiple answers could be selected). 55.3% of all participants selected ‘Made from biomass’, 84.9% ‘biodegradable’, 60.9% ‘recyclable’, and 3.8% ‘Made from petroleum’. This suggests that many participants had incorrect associations with bio-based PMD, despite the brief introduction provided at the start of the section with bio-based items mentioning that bio-based means made from biomass (see Appendix 1.2).

## Discussion

This empirical study provided insight into the public’s preference regarding materials for PMD and its underlying psychological mechanisms. Bio-based PMD were selected as a sustainable alternative to conventional PMD for the purpose of this study, although no such statements were presented to participants. Results showed that the public preferred bio-based over conventional PMD, suggesting that they are open to the implementation of a more sustainable alternative for the materials used for PMD. Since this preference was not strong and differed between PMD used outside or inside the body, implementing such an innovation should be done with care. Especially since unfamiliarity with and misconceptions about bio-based plastic seem to be common, as was observed in the current study and previous research in a consumer context^14,27^. Uncareful introduction could potentially elicit undesirable effects, such as people avoiding health care due to worries based on incorrect assumptions about bio-based plastic (e.g., being biodegradable and/or recyclable). Future research could examine how PMD made from sustainable materials are received by patients and other stakeholders, and optimal ways to implement such innovations. For instance, the sector would likely benefit from an overview of potential challenges that implementation strategies should consider, such as those resulting from misunderstandings about bio-based materials. Additionally, future research could investigate responses to the use of sustainable alternatives for PMD while varying the degree of accompanying information provided to patients (e.g., no accompanying information, a brief explanation, or elaborate accompanying information).

The integral network approach adopted in this study suggests that attitudes toward bio-based PMD are more important for material preference than attitudes toward conventional PMD. This study also showed the importance of emotions for preference for bio-based PMD and attitudes toward PMD in general, both on a network level and more specifically related to preference. Such a central role of emotions toward bio-based plastic is in line with previous research into bio-based plastic in a consumer context^13^. We found positive emotions toward bio-based PMD to be related to preference for bio-based materials for PMD, both used inside and outside of the body. Beliefs about bio-based PMD also played an important role in material preference, although the type of belief that was most relevant differed depending on whether the PMD were used inside or only outside of the body. Note that the cross-sectional design of this study did not allow for inferences about the directions of effects, therefore it remains unknown whether preference is predicted by or predictive of these psychological variables. For instance, emotions about bio-based PMD could predict preference, or preference could predict emotions about bio-based PMD, or both in case of a bidirectional effect. Future longitudinal or experimental research into the direction of effects could shed further light on this. The current study provided an important first step for such future research by demonstrating which variables are (in)directly related to material preferences for different categories of PMD, which could be used to prioritize variables to include in future research.

Distinguishing between relevance subsamples revealed a comparable preference for bio-based plastic for medical devices used inside the body, and a stronger preference for bio-based PMD used outside of the body among recent PMD receivers. Furthermore, the psychological networks of recent PMD receivers and others as potential future receivers were comparable on a global network level. Moreover, although some edges differed significantly between the subsamples, no difference appeared relevant for preference regarding PMD materials. These results suggests that, although including patients remains essential for in-depth insights, insight into (future) PMD receivers’ preferences could be obtained by surveying the public.

Interestingly, the results of this study also suggest that the difference between recent and potential future PMD receivers in preference for bio-based PMD used outside of the body cannot be explained by any of the direct relations between that preference node and other nodes in the network. If this were the case, one would expect to find significant differences between subsamples in edges connected to that preference node. However, an indirect explanation might be provided by the stronger relation between beliefs about the safety of bio-based PMD compared to current PMD and trust in bio-based PMD. That is, thinking bio-based PMD are safer than current PMD might have a stronger indirect effect on preference for bio-based PMD used outside of the body via trust in bio-based PMD in recent PMD receivers. Future research could include additional variables that might explain this difference in preference, that is, variables not included in the current study that might differ between subsamples and are relevant for one’s preference towards the use of bio-based PMD outside of the body. A possible route would be to include variables that might change after being confronted with requiring care, such as perceived dependence on care or maybe even values that underly environmental attitudes, that is, environmental values^28^. Additionally, the cross-sectional design of the current study provides insight in between-person but not within-person effects. Future research could examine whether within-person effects could explain differences between recent and potential future health-care users regarding their preferences for bio-based PMD used outside of the body.

Limitations of this study mainly concern the type of sample (i.e., research panel), which might not be entirely representative of the population, for example because people who report primary education as their educational level tend to be underrepresented in these panels. Also, preferences were measured by self-report, which could provoke socially desirable responses in favor of sustainability. Finally, the survey used in this study was constructed especially for this study and has not been validated.

To conclude, this study suggests there is an openness among the public toward integrating sustainability into material innovations for medical devices and provides a first step toward involving this important stakeholder. Subsequently, it informs those responsible for (sustainable alternatives of) medical devices, such as developers, hospital purchasing departments, or policy makers, about recent and potential future PMD receivers’ perceptions, which is important to incorporate from design to introduction and implementation. Finally, this empirical study shows that receivers’ perspectives should not be seen as a potential barrier, but as additional motivation for transitioning toward sustainable healthcare.

### Supplementary Information

Supplementary Information.

## Data Availability

The datasets generated and analysed during the current study are available in the OSF repository, https://osf.io/5etma/. The R-script for study 2 is also available in the OSF repository.
