# Supplementary material for: Understanding public perceptions toward sustainable healthcare through psychological network analysis of material preference and attitudes toward plastic medical devices
Source: Sci Rep. 2023 Oct 20;13:17938. doi: 10.1038/s41598-023-45172-6 (PMC10589264; doi:10.1038/s41598-023-45172-6)
Supplement: Supplementary file 1 — Supplementary Information. [file 41598_2023_45172_MOESM1_ESM.docx]

Appendix 1

**Understanding public perceptions toward sustainable healthcare through psychological network analysis of material preference and attitudes toward plastic medical devices**

Monique Chambon¹^,^², Janneke E. Elberse¹, Jonas Dalege³, Nick R. M. Beijer¹ & Frenk van Harreveld¹^,^²

¹ National Institute for Public Health and the Environment (RIVM), Antonie van Leeuwenhoeklaan 9, 3721 MA, Bilthoven, The Netherlands

² University of Amsterdam, Department of Social Psychology, Nieuwe Achtergracht 129-B, 1018 WT, Amsterdam, The Netherlands

³ Santa Fe Institute, 1399 Hyde Park Road, Santa Fe, New Mexico 87501, United States of America

Corresponding author:

Monique Chambon

National Institute for Public Health and the Environment (RIVM)

Antonie van Leeuwenhoeklaan 9

3721 MA, Bilthoven, The Netherlands

Email: [monique.chambon@rivm.nl](mailto:monique.chambon@rivm.nl)

# Study 1

**Method**

***Participants and procedure***

A total of 69 participants completed the survey with open-ended questions. Three participants were excluded because of insufficient data quality (i.e., entered random letters or only yes/no). This resulted in a valid *N* = 66. Only participants that met the ‘high relevance’-criteria could complete the survey, meaning that they reported that, in the last two years, they received health care in which plastic medical devices were used (see Appendix 1.2 for specification of criteria and corresponding survey items). The age of the participants ranged from 23 to 79 years (*M* = 52.42, *SD* = 16.32). 40.9% of the participants describes their gender as male (59.1% female, 0% other). 28.8% completed primary education, 36.4% secondary education, and 34.8% higher education.

Participants first provided written consent and then answered questions about demographics and the two relevance-criteria questions. Participants were then asked to list their beliefs (i.e., advantages and disadvantages) and emotions regarding the use of PMD. With each question, the definition of plastic medical devices was presented at the bottom of the page. The order of items on beliefs and emotions was randomized, as was the order of listing advantages and disadvantages within the part on beliefs. The first section provided only general instructions before participants were asked to list their beliefs and emotions; the second section repeated the request to list beliefs and emotions with the instruction to keep in mind the effects of the use of PMD for health, safety, and the environment. Answers were provided in open text fields. Only in the first section were participants forced to enter text in at least two fields for both beliefs and emotions. At the end of the survey, participants could provide additional comments in an open text field.

***Measures***

**General section**

*Introduction*

The following questions are about your thoughts and feelings about using plastic medical devices. We ask separately about your thoughts and your feelings.

Please keep your experience(s) with plastic medical devices in mind throughout the remainder of this study. Also think of the plastic packaging of the devices and plastic protective equipment such as gloves, face masks and aprons.

We repeat the description of plastic medical devices with each question for your reference.

*Instructions*

Your task in the following questions is to write down in a few words or short sentences what comes to mind when you think of the topic plastic medical devices.

- Use as many words or short sentences as you need.

- Mention what comes to mind first and don't think too long about your answers.

- There are no right or wrong answers.

*Beliefs*

These questions are about your thoughts on using plastic medical devices. We ask separately about advantages and disadvantages.

- Advantages of using plastic medical devices are…
  [10 open text fields]
- Disadvantages of using plastic medical devices are…
  [10 open text fields]

*Emotions*

This question is about your feelings about using plastic medical devices. For example, happiness or anger.

- About the use of plastic medical devices, I feel…
  [10 open text fields]

**Health, safety and environment section**

*Instructions*

The following questions are again about your thoughts and feelings about the use of plastic medical devices.

Write down whatever else comes to mind when you specifically think about

the consequences of the use of plastic medical devices for:

- your health and safety, and

- the environment (sustainability).

We repeat this instruction with each question for your reference.

Rewrite a word or short phrase if you are unsure whether you have mentioned it before.

*Beliefs*

Again, these questions are about your thoughts on using plastic medical devices. We ask separately about advantages and disadvantages.

When answering these questions, consider the consequences of using plastic medical devices for:

- Your health and safety: advantages and disadvantages of using plastic medical devices for your health and safety.

- Sustainability: advantages and disadvantages of the use of plastic medical devices for the environment.

- Advantages of using plastic medical devices for health and/or sustainability are…
  [10 open text fields]
- Disadvantages of using plastic medical devices for health and/or sustainability are… [10 open text fields]

*Emotions*

These questions are again about your feelings about using plastic medical devices. For example, happiness or anger.

When answering these questions, consider the consequences of using plastic medical devices for:

- Your health and safety: feelings about using plastic medical devices for your health and safety.

- Sustainability: feelings about the use of plastic medical devices for the environment.

- About the consequences of the use of plastic medical devices for health and/or sustainability, I feel…
  [10 open text fields]

***Analysis***

Analysis of the answers to the open-ended questions consisted of four steps. First, the raw data was cleaned (e.g., removing answers that were unclear or unrelated to the topic). Second, within-person overlap was removed so that every answer was counted once per participant. Such overlap could exist between answers in response to items on beliefs and emotions, or between responses given in the first general section and the second section that specified health, safety and environment. Third, we identified theme’s within the answers to beliefs and emotions. Finally, the frequency of each theme was counted.

**Results**

Table A1.1 provides an overview of the themes as identified in the open-text responses of participants to the thought and emotion listing, including the direction of the theme (positive/negative) in case responses were predominantly unipolar, the corresponding terminology that participants used, and the frequency count of the themes. Participant’s terminology served as input for formulating the corresponding survey items in Study 2. The (Dutch) raw data and more details on the analysis is provided on OSF (https://osf.io/5etma/?view_only=90e51a2e032649fab91b44b778adf740).

Table A1.1 – Themes in open-text responses, including the direction of the theme (positive/negative) and corresponding terminology, and frequency

| Category | Theme/label | Direction and used terminology | Frequency |
| --- | --- | --- | --- |
| Beliefs | Hygiene | Hygienic | 58 |
|  | Ease of use for health-care professionals | Easy to use | 49 |
|  | Duration of use |  | 47 |
|  | Environmental pollution | Bad for the environment | 41 |
|  | Waste (volume) | A lot of waste | 39 |
|  | Properties of material important for devices | Flexible and light | 35 |
|  | Costs | Cheap | 29 |
|  | Availability | (readily) available | 18 |
|  | Waste treatment/degradability | Poorly degradable | 18 |
|  | Recyclable |  | 15 |
|  | Long-term health consequences | Harmful substances or microplastics | 13 |
|  | Quality/reliability |  | 12 |
|  | Short-term health consequences/Safety | Side effects such as an allergic reaction, infection or inflammation | 11 |
|  | Necessity for people's health | Necessary | 10 (+18 with emotions) |
|  | Unnecessary usage | Unnecessary | 7 |
|  | Raw material consumption in production | Lots of raw materials | 3 |
| Emotions | Positive feelings |  | Both general and specific |
|  | Negative feelings |  | Both general and specific |
|  | Safety | Safe | 37 |
|  | Guilt | Guilty | 17 |
|  | Concerned - safety | Concerned | 12 |
|  | Concerned - environmental consequences | '' | '' |
| Involvement | (Un)awareness | Thinking | 11 |

# Survey items

**Relevance**

Participants were first asked to answer questions about their exposure to plastic medical devices to allocate them to the high or low relevance subsample.

Q1. In the past two years, have you been treated by a health-care provider for a physical condition such as an illness or injury or for another medical procedure? For example, in the hospital, emergency room, by home care or by a general practitioner.

Q2. Were plastic medical devices used in this treatment(s)? By plastic medical devices, we mean all plastic devices used by health-care providers.

Examples of plastic medical devices include tubes and bags for administering or draining items such as oxygen, fluids, blood or tube feedings. Other examples include an IV or a catheter.

Answer options for both Q1 and Q2.

1. Yes

2. No

3. Don't know or prefer not to say

Participants who answered ‘Yes’ to both questions formed the high relevance subsample. Participants who answered ‘No’ to either the first or the second question formed the low relevance subsample. Answering ‘Don't know or prefer not to say’ to either question led to exclusion from the survey. The logical expression for this is provided next.

High relevance subsample: Q1 = 1 AND Q2 = 1

Low relevance subsample: Q1 = 2 OR Q2 = 2

Q1 = 3 OR Q2 = 3 🡪 Exclusion

Note that Q2 was not displayed when Q1 was answered with 2 (then allocation to low relevance subsample) or 3 (then exclusion followed).

Table A2.1 – Overview survey items in Study 2.

| **Survey items** | **Answer scale** |
| --- | --- |
| *Section: Current plastic medical devices^a^* | |
| To what extent is the use of plastic medical devices in healthcare a topic that you think about? | Not at all (1) - Very much (7) |
| When you only think about the **positive aspects** and ignore the negative aspects, how positive are you about the use of plastic medical devices in healthcare? | Not at all positive (1) - Very positive (7) |
| When you only think about the **negative aspects** and ignore the positive aspects, how negative are you about the use of plastic medical devices in healthcare? | Not at all negative (1) - Very negative (7) |
| Plastic medical devices are used more than necessary to treat patients. | Strongly disagree (1) - Neutral (4) - Strongly agree (7) |
| Plastic medical devices have important properties. For example, light or flexible. | Strongly disagree (1) - Neutral (4) - Strongly agree (7) |
| Plastic medical devices are readily available for healthcare. | Strongly disagree (1) - Neutral (4) - Strongly agree (7) |
| Plastic medical devices are easy to use for health-care workers. | Strongly disagree (1) - Neutral (4) - Strongly agree (7) |
| Plastic medical devices are… | For single use (1) – Highly reusable (7) |
| Plastic medical devices are… | Not at all recyclable (1) – Highly recyclable (7) |
| Plastic medical devices are… | Not at all of reliable quality (1) – Of highly reliable quality (7) |
| The use of plastic medical devices is hygienic. | Strongly disagree (1) - Neutral (4) - Strongly agree (7) |
| The use of plastic medical devices is necessary to treat patients. | Strongly disagree (1) - Neutral (4) - Strongly agree (7) |
| The use of plastic medical devices is a problem due to the raw materials used in production. | Strongly disagree (1) - Neutral (4) - Strongly agree (7) |
| The use of plastic medical devices is cheap. | Strongly disagree (1) - Neutral (4) - Strongly agree (7) |
| The use of plastic medical devices is bad for the environment. | Strongly disagree (1) - Neutral (4) - Strongly agree (7) |
| The use of plastic medical devices produces a lot of waste. | Strongly disagree (1) - Neutral (4) - Strongly agree (7) |
| The use of plastic medical devices produces waste that is difficult to degrade. | Strongly disagree (1) - Neutral (4) - Strongly agree (7) |
| The use of plastic medical devices is safe for patients. | Strongly disagree (1) - Neutral (4) - Strongly agree (7) |
| The use of plastic medical devices is bad for patients' long-term health. For example, due to harmful substances or microplastics. | Strongly disagree (1) - Neutral (4) - Strongly agree (7) |
| When I think about the use of plastic medical devices, I have positive feelings. For example happy or satisfied. | Strongly disagree (1) - Strongly agree (7) |
| When I think about the use of plastic medical devices, I have negative feelings. For example sad or angry. | Strongly disagree (1) - Strongly agree (7) |
| When I think about the use of plastic medical devices, I feel safe. | Strongly disagree (1) - Strongly agree (7) |
| When I think about the use of plastic medical devices, I feel guilty. | Strongly disagree (1) - Strongly agree (7) |
| When I think about the use of plastic medical devices, I feel concerned about the patient's health. | Strongly disagree (1) - Strongly agree (7) |
| When I think about the use of plastic medical devices, I feel concerned about the environment. | Strongly disagree (1) - Strongly agree (7) |
| I think other people favor the use of plastic medical devices in healthcare. | Strongly disagree (1) - Neutral (4) - Strongly agree (7) |
| I think I have influence over the use of plastic medical devices. | Strongly disagree (1) - Neutral (4) - Strongly agree (7) |
| I trust manufacturers of plastic medical devices. | Strongly disagree (1) - Neutral (4) - Strongly agree (7) |
| I trust laws and regulations about plastic medical devices. | Strongly disagree (1) - Neutral (4) - Strongly agree (7) |
| I trust choices made in healthcare about plastic medical devices. | Strongly disagree (1) - Neutral (4) - Strongly agree (7) |
| *Section: bio-based plastic medical devices^b^* | |
| When you only think about the **positive aspects** and ignore the negative aspects, how positive are you about the use of bio-based plastic medical devices in healthcare? | Not at all positive (1) - Very positive (7) |
| When you only think about the **negative aspects** and ignore the positive aspects, how negative are you about the use of bio-based plastic medical devices in healthcare? | Not at all negative (1) - Very negative (7) |
| Bio-based plastic medical devices… | Are for single use (1) – Can be used very often (7) |
| The use of bio-based plastic medical devices is, compared to regular plastic medical devices,… | Much less hygienic (1) - No difference (4) – Much more hygienic (7)^c^ |
| The use of bio-based plastic medical devices is, compared to regular plastic medical devices,… | Much worse due to the raw materials used in production (1) - No difference (4) - Much better due to the raw materials used in production (7)^c^ |
| The use of bio-based plastic medical devices is, compared to regular plastic medical devices,… | Much worse for the environment (1) - No difference (4) - Much better for the environment (7)^c^ |
| The use of bio-based plastic medical devices is, compared to regular plastic medical devices,… | Much less safe for patients (1) - No difference (4) - Much safer for patients (7)^c^ |
| The use of bio-based plastic medical devices is, compared to regular plastic medical devices,… | Much worse for long-term health (1) - No difference (4) - Much better for long-term health (7)^c^ |
| The use of bio-based plastic medical devices produces, compared to regular plastic medical devices,… | Much less waste (1) - No difference (4) - Much more waste (7)^c^ |
| The use of bio-based plastic medical devices produces, compared to regular plastic medical devices,… | Waste that is more difficult to degrade (1) - No difference (4) - Waste that is more easily degradable (7)^c^ |
| Bio-based plastic medical devices are, compared to regular plastic medical devices,… | Much less recyclable (1) - No difference (4) - Much more recyclable (7)^c^ |
| Bio-based plastic medical devices are, compared to regular plastic medical devices,… | Of much worse quality (1) - No difference (4) – Of much better quality (7)^c^ |
| When I think about the use of bio-based plastic medical devices, compared to regular plastic medical devices, I have... | Much less positive feelings (such as happy or satisfied) (1) - No difference (4) - Much more positive feelings (such as happy or satisfied) (7)^c^ |
| When I think about the use of bio-based plastic medical devices, compared to regular plastic medical devices, I have... | Much less negative feelings (such as sad or angry) (1) - No difference (4) - Much more negative feelings (such as sad or angry) (7)^c^ |
| About the use of bio-based plastic medical devices, compared to regular plastic medical devices, I feel,… | Much less safe (1) - No difference (4) -Much safer (7)^c^ |
| About the use of bio-based plastic medical devices, compared to regular plastic medical devices, I feel,… | Much less guilty (1) - No difference (4) -Much more guilty (7)^c^ |
| About the use of bio-based plastic medical devices, compared to regular plastic medical devices, I feel,… | Much less concerned about the health of patients (1) - No difference (4) - Much more concerned about the health of patients (7)^c^ |
| About the use of bio-based plastic medical devices, compared to regular plastic medical devices, I feel,… | Much less concerned about the environment (1) - No difference (4) - Much more concerned about the environment (7)^c^ |
| I think other people favor the use of bio-based plastic medical devices in healthcare. | Strongly disagree (1) - Neutral (4) - Strongly agree (7) |
| I think I have influence over the use of bio-based plastic medical devices. | Strongly disagree (1) - Neutral (4) - Strongly agree (7) |
| I trust manufacturers of bio-based plastic medical devices. | Strongly disagree (1) - Neutral (4) - Strongly agree (7) |
| I trust laws and regulations about bio-based plastic medical devices. | Strongly disagree (1) - Neutral (4) - Strongly agree (7) |
| I trust choices made in healthcare about bio-based plastic medical devices. | Strongly disagree (1) - Neutral (4) - Strongly agree (7) |
| *Preference* - Imagine that for the following products you get to choose between current plastic medical devices and bio-based plastic medical devices. Which would you choose? | |
| Plastic medical devices that do *not* come into contact with the body. For example, bags in which urine is collected or packing material. | Definitely current plastic (1) - No preference (4) - Definitely bio-based plastic (7) |
| Plastic medical devices that come into contact with the *outside* of the body. For example stoma bags or gloves. | Definitely current plastic (1) - No preference (4) - Definitely bio-based plastic (7) |
| Plastic medical devices that come in contact with the *inside* of the body. For example, a catheter or tube feeding set. | Definitely current plastic (1) - No preference (4) - Definitely bio-based plastic (7) |
| *Health* |  |
| How is your health in general? | Very poor (1) - Very good (7) |
| In the past two years, how often have you come into contact with plastic medical devices? | Rarely or never (1); Annually (2); Every six months (3); Monthly (4); Weekly (5); Several times a week (6); Daily (7) |

^a^ Section introduction – current plastic medical devices: This questionnaire is about your thoughts and feelings about the use of plastic medical devices. With plastic medical devices we mean all plastic devices used by health-care providers. Examples of plastic medical devices include tubes and bags for administering or draining items such as oxygen, fluids, blood or tube feedings. Other examples include an IV or a catheter. Also consider plastic packaging of medical devices and plastic protective equipment such as gloves, face masks and aprons. Want to read back this description? Then place your mouse over the words "plastic medical devices" in the questionnaire. This only works on a computer. There are no right or wrong answers: choose the answer that best represents your opinion or feelings.
^b^ Section introduction – bio-based plastic medical devices: The following questions are about your thoughts and feelings about "bio-based" plastic medical devices. Bio-based plastic is made from biomass (usually plants). For example, bio-based plastic can be made from corn or sugarcane. The difference between bio-based and regular plastic medical devices is the material they are made from: bio-based plastic medical devices are made from biomass and regular plastic medical devices are made from petroleum. Imagine all plastic medical devices in healthcare are made of bio-based plastic when answering the following questions. Bio-based plastic medical devices are not used in healthcare today, but they may be in the future. Therefore, we would like to better understand what bio-based plastic medical devices evoke in people. There are no right or wrong answers: choose the answer that best represents your opinion or feelings.
^c^ Note that scale for respondents ranged from -3 to 0 to +3, respectively.

**Other survey items**

- Consent
- Demographics (age, gender, education, residential area)
- Bio-based check question
  - What characteristics do you think of when you think of bio-based plastic medical devices? It is possible to give multiple answers.
    - Made from petroleum
    - Made from biomass
    - Biodegradable
    - Recyclable
    - Other, namely..
- Attention checks (two items, answering both items wrong led to exclusion of the survey)
  - To ensure that you keep your attention during this survey, we ask that you select "Strongly agree" [/"Strongly disagree"] here.

# Variable construction

Table A3.1 lists the variables in the network and their construction method. For the variable construction, we first excluded items from predetermined variables (see Appendix 1.2 for corresponding items). We then conducted dimension reduction analyses, more specifically Principal Axis Factoring (PAF), to identify components in the data that formed separate variables. This was conducted with the dataset from the total sample, and separately for items on beliefs and emotions for both current and bio-based plastic medical devices. The PAF results are presented below. The selected rotation method was Oblimin due to the expected intercorrelation between items. Extraction of dimensions was based on eigenvalues greater than one.

Table A3.1 - Overview of variables and their construction method.

| **Variable** | **Approach to combining items in variable** |
| --- | --- |
| *General* |  |
| Age | Predetermined single item variable |
| Health | Predetermined single item variable |
| Exposure | Predetermined single item variable |
| Thinking | Predetermined single item variable |
| Preference External | Combined 2 item variable |
| Preference Internal | Predetermined single item variable |
| *Both current and bio-based* |  |
| [Current/Bio]_Ambivalence | Predetermined 2 item construct calculated with formula |
| [Current/Bio]_PerceivedControl | Predetermined single item variable |
| [Current/Bio]_SocialNorm | Predetermined single item variable |
| Trust | Predetermined 3 item construct (PAF confirmed single component) |
|  |  |
| *Current* |  |
| Current_BeliefsNegative | PAF – Component identified in items on beliefs |
| Current_BeliefsPositive | PAF – Component identified in items on beliefs |
| Current_BeliefsRecycle | PAF – Component identified in items on beliefs, resulted in single item variable |
| Current_BeliefsUse | PAF – Component identified in items on beliefs, resulted in single item variable |
| Current_EmotionNegative | PAF – Component identified in items on emotions |
| Current_EmotionPositive | PAF – Component identified in items on emotions |
|  |  |
| *Bio-based* |  |
| Bio_BeliefsSafety | PAF – Component identified in items on beliefs |
| Bio_BeliefsEnvironment | PAF – Component identified in items on beliefs |
| Bio_BeliefsUse | PAF – Component identified in items on beliefs, resulted in single item variable |
| Bio_BeliefHealthLongterm | PAF – Component identified in items on beliefs, resulted in single item variable |
| Bio_Emotions | PAF – Single component |

**Predetermined combined variables**

*Preference*

Preference regarding current or bio-based PMD was surveyed with three items, that is, for PMD that a) do *not* come into contact with the body, b) come into contact with the *outside* of the body, and c) come into contact with the *inside* of the body. The first two preference items were highly correlated (*r* = .77) and therefore combined into one variable (i.e., *PreferenceExternal; a* = .87). This optimizes the balance between clarity and nuance: including all preference items as separate nodes complicates the interpretation of the network, whereas combining them into one variable reduces nuance. Table A3.2 shows the final variables related to preference including the survey items. Higher scores on these variables indicates a preference for bio-based plastic medical devices (i.e., a score below 4 indicating a preference for current plastic medical devices and a score above 4 indicating a preference for bio-based plastic medical devices). Note that sensitivity analyses were conducted to evaluate how the main results are affected when combining all three preference items into one preference node (*a* = .82), and when including all three preference items as single item nodes.

Table A3.2 – Survey items for both variables related to preference

| **Variable** | **Survey items** | **Item label dataset** |
| --- | --- | --- |
| Preference External (*a* = .87) | Plastic medical devices that do *not* come into contact with the body. For example, bags in which urine is collected or packing material. | Pref_1 |
|  | Plastic medical devices that come into contact with the *outside* of the body. For example stoma bags or gloves. | Pref_2 |
| Preference Internal | Plastic medical devices that come in contact with the *inside* of the body. For example, a catheter or tube feeding set. | Pref_3 |

Note. Introduction: Imagine that for the following products you get to choose between current plastic medical devices and bio-based plastic medical devices. Which would you choose? Scale: Definitely current plastic (1) - No preference (4) - Definitely bio-based plastic (7).

*Ambivalence – Variables Current_Ambivalence and Bio_ Ambivalence*

The variables on ambivalence (i.e., *Current_Ambivalence* and *Bio_Ambivalence*) were calculated with the formula (P + N)/2 - |P – N|, that is, subtract the absolute difference between the positive and negative item from the average of both, ^1^ with the item for P being ‘*When you only think about the positive aspects and ignore the negative aspects, how positive are you about the use of [bio-based] plastic medical devices in healthcare*?’, and for N being ‘*When you only think about the negative aspects and ignore the positive aspects, how negative are you about the use of [bio-based] plastic medical devices in healthcare?*’ cf.^2^ This resulted in a score between -2 and 7. To increase this variable’s conformance with the other variables, we then added 3 to the score so that scores ranged from 1 to 10 with a higher score indicating more ambivalence.

*Trust – Variables Current_Trust and Bio_Trust*

This PAF analysis included three items on trust related to plastic medical devices (i.e., trust in producers, regulations, and choices made in healthcare; see Table A3.3). Kaiser-Meyer-Olkin Measure of Sampling Adequacy was .72 (bio-based .73). Results suggested one component with the three trust items for both current and bio-based items. This resulted in the variables *Current_Trust* (*a* = .82) and *Bio_Trust* (*a* = .86) with the three items shown below, with a higher score indicating more trust.

Table A3.3 – Survey items with PAF component loads for items on trust related to current and bio-based plastic medical devices.

| **No.** | **Survey items** | **Current_Trust  Component load** | **Item label dataset** | **Bio_Trust  Component load** | **Item label dataset** |
| --- | --- | --- | --- | --- | --- |
| 1 | I trust manufacturers of [bio-based] plastic medical devices. | .78 | Gen_Oth_3 | .83 | Bio_Oth_3 |
| 2 | I trust laws and regulations about [bio-based] plastic medical devices. | .76 | Gen_Oth_4 | .85 | Bio_Oth_4 |
| 3 | I trust choices made in healthcare about [bio-based] plastic medical devices. | .80 | Gen_Oth_5 | .78 | Bio_Oth_5 |

**Component analysis beliefs**

*Beliefs current plastic medical devices*

This PAF analysis included 16 items on beliefs related to current plastic medical devices (see Table A3.4). Kaiser-Meyer-Olkin Measure of Sampling Adequacy was .86. Initial results identified three components within all items on beliefs related to current plastic medical devices, that is, positive beliefs (V1), negative beliefs (V2) and durability (V3). Results are discussed in detail below.

Table A3.4 – Survey items with PAF component loads for items on beliefs related to current plastic medical devices. Values below the absolute value of .25 are omitted for readability.

| **No.** | **Survey items** |  | **V1** | **V2** | **V3** | **Item label dataset** |
| --- | --- | --- | --- | --- | --- | --- |
| 1 | Plastic medical devices have important properties. For example, light or flexible. |  | -0.69 |  |  | Gen_Cog_2 |
| 2 | Plastic medical devices are readily available for healthcare. |  | -0.64 |  |  | Gen_Cog_3 |
| 3 | Plastic medical devices are easy to use for health-care workers. |  | -0.78 |  |  | Gen_Cog_4 |
| 4 | Plastic medical devices are… Not at all of reliable quality (1) – Of highly reliable quality (7) |  | -0.58 |  |  | Gen_Cog_7 |
| 5 | The use of plastic medical devices is hygienic. |  | -0.63 |  |  | Gen_Cog_8 |
| 6 | The use of plastic medical devices is necessary to treat patients. |  | -0.46 | 0.33 |  | Gen_Cog_9 |
| 7 | The use of plastic medical devices is cheap. |  | -0.35 |  |  | Gen_Cog_11 |
| 8 | The use of plastic medical devices is safe for patients. |  | -0.62 |  |  | Gen_Cog_15 |
| 9 | Plastic medical devices are used more than necessary to treat patients. |  |  | -0.63 |  | Gen_Cog_1 |
| 10 | The use of plastic medical devices is a problem due to the raw materials used in production. |  |  | -0.66 |  | Gen_Cog_10 |
| 11 | The use of plastic medical devices is bad for the environment. |  |  | -0.7 |  | Gen_Cog_12 |
| 12 | The use of plastic medical devices produces a lot of waste. |  |  | -0.63 |  | Gen_Cog_13 |
| 13 | The use of plastic medical devices produces waste that is difficult to degrade. |  |  | -0.65 |  | Gen_Cog_14 |
| 14 | The use of plastic medical devices is bad for patients' long-term health. For example, due to harmful substances or microplastics. |  |  | -0.61 |  | Gen_Cog_16 |
| 15 | Plastic medical devices are… Not at all recyclable (1) – Highly recyclable (7) |  | -0.28 |  | -0.34 | Gen_Cog_6 |
| 16 | Plastic medical devices are… For single use (1) – Highly reusable (7) |  |  |  | -0.47 | Gen_Cog_5 |

- *Positive beliefs (V1) – Variable Current_BeliefsPositive*

PAF results showed that multiple items on positive beliefs formed one component (see Table A3.4). Although item 7 showed a rather low component load, excluding the item did not improve scale reliability considerably (a = .81, if deleted a = .83), therefore the item was maintained. Furthermore, although item 6 loads on two components (V1 and V2), it is included in component V1 because of a higher component load and a decrease in alpha if excluded (*a* = .81, if deleted *a* = .80). Finally, although item 15 showed component loads on both V1 and V3, the load was higher for V3 and including this item in V1 would decrease scale reliability (*a* = .81, if included *a* = .77) and was therefore not included in this component. This resulted in the variable *Current_BeliefsPositive* (*a* = .81) with the eight items shown in Table A3.5, with a higher score indicating more positive beliefs about current plastic medical devices.

Table A3.5 – Survey items with PAF component loads for items on positive beliefs about current plastic medical devices. Component loads are based on PAF shown in Table A3.4.

| **No.** | **Survey items** |  | **Current_BeliefsPositive Component load** | **Item label dataset** |
| --- | --- | --- | --- | --- |
| 1 | Plastic medical devices have important properties. For example, light or flexible. |  | -0.69 | Gen_Cog_2 |
| 2 | Plastic medical devices are readily available for healthcare. |  | -0.64 | Gen_Cog_3 |
| 3 | Plastic medical devices are easy to use for health-care workers. |  | -0.78 | Gen_Cog_4 |
| 4 | Plastic medical devices are… Not at all of reliable quality (1) – Of highly reliable quality (7) |  | -0.58 | Gen_Cog_7 |
| 5 | The use of plastic medical devices is hygienic. |  | -0.63 | Gen_Cog_8 |
| 6 | The use of plastic medical devices is necessary to treat patients. |  | -0.46 | Gen_Cog_9 |
| 7 | The use of plastic medical devices is cheap. |  | -0.35 | Gen_Cog_11 |
| 8 | The use of plastic medical devices is safe for patients. |  | -0.62 | Gen_Cog_15 |

- *Negative beliefs (V2) – Variable* *Current_BeliefsNegative*

Results also showed that multiple items on negative beliefs formed one component (see in Table A3.4). As discussed above, item 6 showed a component load on both V1 and this component, but was included in V1. This resulted in the variable *Current_BeliefsNegative* (*a* = .82) with the six items shown at in Table A3.6, with a higher score indicating more negative beliefs about current plastic medical devices.

Table A3.6 – Survey items with PAF component loads for items on negative beliefs about current plastic medical devices. Component loads are based on PAF shown in Table A3.4.

| **No.** | **Survey items** |  | **Current_BeliefsNegative Component load** | **Item label dataset** |  |
| --- | --- | --- | --- | --- | --- |
| 1 | Plastic medical devices are used more than necessary to treat patients. |  | -0.63 | Gen_Cog_1 |  |
| 2 | The use of plastic medical devices is a problem due to the raw materials used in production. |  | -0.66 | Gen_Cog_10 |  |
| 3 | The use of plastic medical devices is bad for the environment. |  | -0.7 | Gen_Cog_12 |  |
| 4 | The use of plastic medical devices produces a lot of waste. |  | -0.63 | Gen_Cog_13 |  |
| 5 | The use of plastic medical devices produces waste that is difficult to degrade. |  | -0.65 | Gen_Cog_14 |  |
| 6 | The use of plastic medical devices is bad for patients' long-term health. For example, due to harmful substances or microplastics. |  | -0.61 | Gen_Cog_16 |  |

- *Durability (V3) – Variables* *Current_BeliefRecycle and Current_BeliefUse*

Finally, results showed that two items on positive beliefs formed one component (see item 15 and 16 in Table A3.4). As discussed, although item 15 also showed a component load on *Current_BeliefsPositive,* the item was not included in that variable. However, the scale reliability of V3 was low (*a* = .35), as was the correlation between the two items (*r* = .21). The items were therefore not combined into one variable, but because of their expected relevance for the network they were included as separate single item variables: *Current_BeliefRecycle* (item 15), with higher scores indicating the belief that current plastic medical devices are recyclable, and *Current_BeliefUse* (item 16), with higher scores indicating the belief that current plastic medical devices can be reused (i.e., score below 4 indicates one tends to believe current plastic medical devices are for single use, and score above 4 indicates one tends to believe current plastic medical devices can be reused).

*Beliefs bio-based plastic medical devices*

This PAF analysis included ten items on beliefs related to bio-based plastic medical devices (see Table A3.7). Kaiser-Meyer-Olkin Measure of Sampling Adequacy was .80. Initial results identified two components within all items on beliefs related to bio-based plastic medical devices, that is, safety beliefs (V1) and environmental beliefs (V2). Results are discussed in detail below.

Table A3.7 – Survey items with PAF component loads for items on beliefs related to bio-based plastic medical devices. Values below the absolute value of .25 are omitted for readability.

| **No.** | **Survey items** |  | **V1** |  | **V2** | **Item label dataset** |
| --- | --- | --- | --- | --- | --- | --- |
| 1 | *Bio-based* plastic medical devices… Are for single use (1) – Can be used very often (7) |  | -0.41 |  |  | Bio_Cog_1 |
| 2 | The use of *bio-based* plastic medical devices is, compared to regular plastic medical devices,… Much less hygienic (1) - No difference (4) – Much more hygienic (7)^a^ |  | -0.68 |  |  | Bio_Cog_2 |
| 3 | The use of *bio-based* plastic medical devices is, compared to regular plastic medical devices,… Much less safe for patients (1) - No difference (4) - Much safer for patients (7)^a^ |  | -0.7 |  |  | Bio_Cog_5 |
| 4 | *Bio-based* plastic medical devices are, compared to regular plastic medical devices,… Of much worse quality (1) - No difference (4) – Of much better quality (7)^a^ |  | -0.75 |  |  | Bio_Cog_10 |
| 5 | The use of *bio-based* plastic medical devices is, compared to regular plastic medical devices,… Much worse due to the raw materials used in production (1) - No difference (4) - Much better due to the raw materials used in production (7)^a^ |  |  |  | -0.69 | Bio_Cog_3 |
| 6 | The use of *bio-based* plastic medical devices is, compared to regular plastic medical devices,… Much worse for the environment (1) - No difference (4) - Much better for the environment (7)^a^ |  |  |  | -0.79 | Bio_Cog_4 |
| 7 | The use of *bio-based* plastic medical devices produces, compared to regular plastic medical devices,… Much less waste (1) - No difference (4) - Much more waste (7)^a^ |  |  |  | 0.33 | Bio_Cog_7 |
| 8 | The use of *bio-based* plastic medical devices produces, compared to regular plastic medical devices,… Waste that is more difficult to degrade (1) - No difference (4) - Waste that is more easily degradable (7)^a^ |  |  |  | -0.8 | Bio_Cog_8 |
| 9 | *Bio-based* plastic medical devices are, compared to regular plastic medical devices,… Much less recyclable (1) - No difference (4) - Much more recyclable (7)^a^ |  |  |  | -0.7 | Bio_Cog_9 |
| 10 | The use of *bio-based* plastic medical devices is, compared to regular plastic medical devices,… Much worse for long-term health (1) - No difference (4) - Much better for long-term health (7)^a^ |  | -0.37 |  | -0.4 | Bio_Cog_6 |

^a^ Note that scale for respondents ranged from -3 to 0 to +3, respectively.

- *Safety beliefs (V1) – Variables* *Bio_BeliefsSafe and* *Bio_BeliefUse*

Results showed that multiple items on safety beliefs about bio-based plastic medical devices formed one component (see Table A3.7). Item 1 showed a rather low component load and excluding the item resulted in a considerable improvement in scale reliability (analysis with item 1 – 4; *a* = .65, if deleted *a* = .77). This item was therefore excluded from this component and included as a single item variable given its expected relevance for the network (*Bio_BeliefUse*). Higher scores on this variable indicate stronger beliefs that bio-based plastic medical devices can be reused (i.e., score below 4 indicates one tends to believe bio-based plastic medical devices are for single use, and score above 4 indicates one tends to believe bio-based plastic medical devices can be reused). Item 10 loads on both components, but is not included in this component since it would slightly decrease scale reliability (without *a* = .77, with *a* = .75). The remaining items in this component formed the variable *Bio_BeliefsSafe* (*a* = .77) consisting of the three items shown in Table A3.8, with a higher score indicating stronger beliefs that bio-based plastic medical devices are safer than current plastic medical devices. That is, a score below 4 indicates one tends to believe that bio-based plastic medical devices are less safe than current plastic medical devices, and a score above 4 indicates one tends to believe bio-based plastic medical devices are safer than current plastic medical devices.

Table A3.8 – Survey items with PAF component loads for items on safety beliefs about bio-based plastic medical devices. Component loads are based on PAF shown in Table A3.7.

| **No.** | **Survey items** |  | **Bio_BeliefsSafe Component load** | **Item label dataset** |
| --- | --- | --- | --- | --- |
| 1 | The use of *bio-based* plastic medical devices is, compared to regular plastic medical devices,… Much less hygienic (1) - No difference (4) – Much more hygienic (7)^a^ |  | -0.68 | Bio_Cog_2 |
| 2 | The use of *bio-based* plastic medical devices is, compared to regular plastic medical devices,… Much less safe for patients (1) - No difference (4) - Much safer for patients (7)^a^ |  | -0.7 | Bio_Cog_5 |
| 3 | *Bio-based* plastic medical devices are, compared to regular plastic medical devices,… Of much worse quality (1) - No difference (4) – Of much better quality (7)^a^ |  | -0.75 | Bio_Cog_10 |

^a^ Note that scale for respondents ranged from -3 to 0 to +3, respectively.

- *Environmental beliefs (V2) – Variable* *Bio_BeliefsEnviron and Bio_BeliefHealthLongterm*

Results showed that multiple items on environmental and health beliefs about bio-based plastic medical devices formed one component (see Table A3.7). Item 7 showed a rather low component load but excluding it did not result in a considerable improvement in scale reliability thus the item was maintained (analysis with item 5 – 9; *a* = .80, if deleted *a* = .84). As discussed, item 10 also loads on this component. Including this item does not improve scale reliability (remains *a* = .80) and would impede its interpretation (i.e., would result in a variable with five environmental and one long term health item). Thus, since item 10 has a comparable load on both components but does not contribute to either variable, this item is included as a single item variable (*Bio_BeliefHealthLongterm*). Higher scores on this variable indicate stronger beliefs that bio-based plastic medical devices are better for one’s health in the long term than current plastic medical devices (i.e., score below 4 indicates one tends to believe bio-based plastic medical devices are worse than current plastic medical devices for one’s health on the long term, and score above 4 indicates one tends to believe bio-based plastic medical are better than current plastic medical devices for one’s health in the long term). The remaining items in this component formed the variable *Bio_BeliefsEnviron* (*a* = .80) with the five items shown in Table A3.9, with a higher score indicating stronger beliefs that bio-based plastic medical devices are better for the environment than current plastic medical devices. That is, a score below 4 indicates one tends to believe that bio-based plastic medical devices are worse for the environment than current plastic medical devices, and a score above 4 indicates one tends to believe bio-based plastic medical devices are better for the environment than current plastic medical devices.

Table A3.9 – Survey items with PAF component loads for items on environmental beliefs about bio-based plastic medical devices. Component loads are based on PAF shown in Table A3.7.

| **No.** | **Survey items** |  | **Bio_BeliefsEnviron Component load** | **Item label dataset** |
| --- | --- | --- | --- | --- |
| 1 | The use of *bio-based* plastic medical devices is, compared to regular plastic medical devices,… Much worse due to the raw materials used in production (1) - No difference (4) - Much better due to the raw materials used in production (7)^a^ |  | -0.69 | Bio_Cog_3 |
| 2 | The use of *bio-based* plastic medical devices is, compared to regular plastic medical devices,… Much worse for the environment (1) - No difference (4) - Much better for the environment (7)^a^ |  | -0.79 | Bio_Cog_4 |
| 3 | The use of *bio-based* plastic medical devices produces, compared to regular plastic medical devices,… Much less waste (1) - No difference (4) - Much more waste (7)^a,b^ |  | 0.33 | Bio_Cog_7 |
| 4 | The use of *bio-based* plastic medical devices produces, compared to regular plastic medical devices,… Waste that is more difficult to degrade (1) - No difference (4) - Waste that is more easily degradable (7)^a^ |  | -0.8 | Bio_Cog_8 |
| 5 | *Bio-based* plastic medical devices are, compared to regular plastic medical devices,… Much less recyclable (1) - No difference (4) - Much more recyclable (7)^a^ |  | -0.7 | Bio_Cog_9 |

^a^ Note that scale for respondents ranged from -3 to 0 to +3, respectively.
^b^ This item was reverse coded before combining into a variable for consistent interpretation.

**Component analysis emotions**

*Emotions current plastic medical devices*

This PAF analysis included six items on emotions related to current plastic medical devices (see Table A3.10). Kaiser-Meyer-Olkin Measure of Sampling Adequacy was .76. Results suggested two components within these emotion items, that is, positive emotions (V1) and negative emotions (V2). Results are discussed in detail below.

Table A3.10 – Survey items with PAF component loads for items on emotions related to current plastic medical devices. Values below the absolute value of .25 are omitted for readability.

| **No.** | **Survey items** | **V1** | **V2** | **Item label dataset** |
| --- | --- | --- | --- | --- |
| 1 | When I think about the use of plastic medical devices, I have positive feelings. For example happy or satisfied. | 1.74 |  | Gen_Emo_1 |
| 2 | When I think about the use of plastic medical devices, I have negative feelings. For example sad or angry. |  | -0.73 | Gen_Emo_2 |
| 3 | When I think about the use of plastic medical devices, I feel safe.^a^ |  | 0.41 | Gen_Emo_3 |
| 4 | When I think about the use of plastic medical devices, I feel guilty. |  | -0.8 | Gen_Emo_4 |
| 5 | When I think about the use of plastic medical devices, I feel concerned about the patient's health. |  | -0.64 | Gen_Emo_5 |
| 6 | When I think about the use of plastic medical devices, I feel concerned about the environment. |  | -0.64 | Gen_Emo_6 |

^a^ This item was reverse coded before combining into a variable for consistent interpretation.

- *Positive emotions (V1) - Variable* *Current_EmotionPositive*

This single item variable consists of item 1 in Table A3.10. Higher scores indicate more positive emotions related to current plastic medical devices.

- *Negative emotions (V2)* – *Variable* *Current_EmotionNegative*

PAF results showed that five items on negative emotions about current plastic medical devices formed one component (see item 2 – 6 in Table A3.10). Although item 3 showed a rather low component load, excluding the item did not improve scale reliability considerably (a = .78, if deleted a = .79), therefore the item was maintained. This resulted in the variable *Current_EmotionNegative* (a = .78), with higher scores indicating more negative emotions related to current plastic medical devices.

*Emotions bio-based plastic medical devices*

This PAF analysis included six items on emotions related to bio-based plastic medical devices (see Table A3.11). Kaiser-Meyer-Olkin Measure of Sampling Adequacy was .80. Results suggested one component with every items on emotions about bio-based plastic medical devices. Although excluding item 3 would increase scale reliability (*a* = .84, if deleted *a* = .85), the item was maintained because deletion would not improve scale reliability considerably and the PAF results suggested one component.

Table A3.11 – Survey items with PAF component loads for items on emotions related to bio-based plastic medical devices.

| **No.** | **Survey items** | **Bio_Emotions Component load** | **Item label dataset** |
| --- | --- | --- | --- |
| 1 | When I think about the use of *bio-based* plastic medical devices, compared to regular plastic medical devices, I have... Much less positive feelings (such as happy or satisfied) (1) - No difference (4) - Much more positive feelings (such as happy or satisfied) (7)^a^ | 0.74 | Bio_Emo_1 |
| 2 | When I think about the use of *bio-based* plastic medical devices, compared to regular plastic medical devices, I have... Much less negative feelings (such as sad or angry) (1) - No difference (4) - Much more negative feelings (such as sad or angry) (7)^a,b^ | -0.77 | Bio_Emo_2 |
| 3 | About the use of *bio-based* plastic medical devices, compared to regular plastic medical devices, I feel,… Much less safe (1) - No difference (4) - Much safer (7)^a^ | 0.39 | Bio_Emo_3 |
| 4 | About the use of *bio-based* plastic medical devices, compared to regular plastic medical devices, I feel,… Much less guilty (1) - No difference (4) - Much more guilty (7)^a,b^ | -0.80 | Bio_Emo_4 |
| 5 | About the use of *bio-based* plastic medical devices, compared to regular plastic medical devices, I feel,… Much less concerned about the health of patients (1) - No difference (4) - Much more concerned about the health of patients (7)^a,b^ | -0.62 | Bio_Emo_5 |
| 6 | About the use of *bio-based* plastic medical devices, compared to regular plastic medical devices, I feel,… Much less concerned about the environment (1) - No difference (4) - Much more concerned about the environment (7)^a,b^ | -0.72 | Bio_Emo_6 |

^a^ Note that scale for respondents ranged from -3 to 0 to +3, respectively.
^b^ This item was reverse coded before combining into a variable for consistent interpretation.

# Edge accuracy and difference

A PDF-file with information on the edge accuracy (i.e., confidence intervals for each edge) and the results of edge difference tests can be found on OSF (https://osf.io/5etma/?view_only=90e51a2e032649fab91b44b778adf740). Edges that differ significantly at *a* = .05 are indicated with black boxes. See ^3^ for more information on accuracy analyses and difference tests.

# Node strength values, stability and difference

**Node strength values**

Table A5.1 – Node strength values of the total sample.

| Variables | Total |
| --- | --- |
| Age | 0.23 |
| **Bio_AmbiDef** | **0.82** |
| Bio_BelEnvir | 1.22 |
| Bio_BelHealLong | 0.74 |
| **Bio_BelSafe** | **0.97** |
| Bio_BelUse | 0.83 |
| Bio_Control | 0.84 |
| Bio_Emo | 1.34 |
| Bio_Norm | 0.61 |
| Bio_Trust | 1.07 |
| Cur_AmbiDef | 0.56 |
| Cur_BelNeg | 1.18 |
| Cur_BelPos | 1.24 |
| Cur_BelRecy | 0.64 |
| Cur_BelUse | 0.63 |
| Cur_Control | 0.84 |
| **Cur_EmoNeg** | **1.56** |
| Cur_EmoPos | 0.78 |
| Cur_Norm | 0.38 |
| Cur_Think | 0.82 |
| Cur_Trust | 1.28 |
| Exposure | 0.63 |
| **Health** | **0.39** |
| PrefExt | 1.06 |
| PrefInt | 0.89 |

**Node stability and difference**

A PDF-file with information on the node stability and the results of node strength difference tests can be found on OSF (https://osf.io/5etma/?view_only=90e51a2e032649fab91b44b778adf740). Nodes that differ significantly at *a* = .05 are indicated with black boxes. Values in the diagonal boxes are node strength values. See ^3^ for more information on stability analysis and difference tests.

# Comparing relevance subsamples


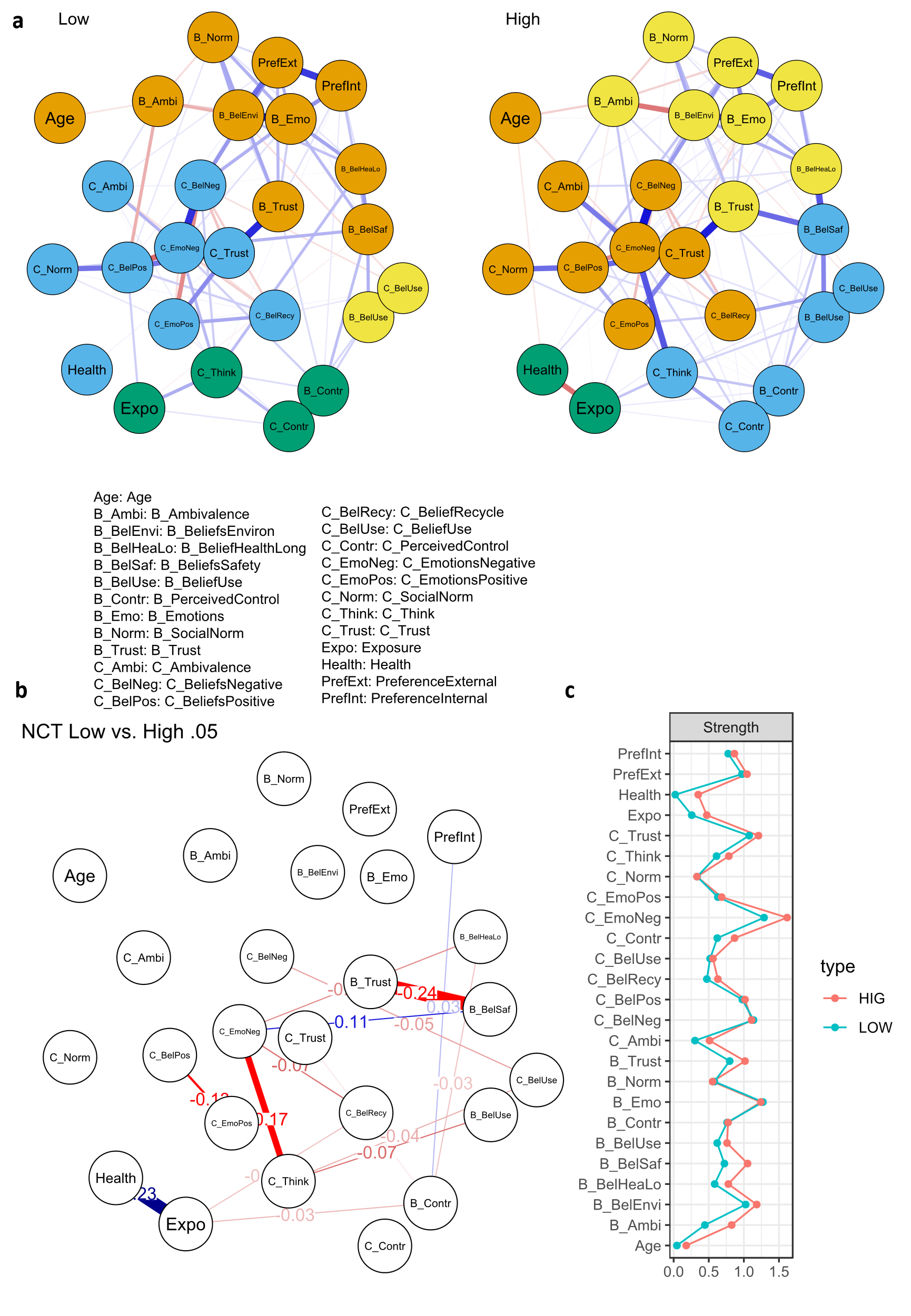


Figure A6.1 - a) Psychological network toward plastic medical devices for the low relevance subsample (left) and the high relevance subsample (right). See Figure 1a in the main text for interpretation guidelines and Table A6.1 for corresponding edge weights); b) Results of the Network Comparison Test (NCT), shows edges that differ significantly between the relevance subsamples with *p*-value < .05. The magnitude of the edge differences is indicated by edge width. A blue (red) edge indicates that the relation in the High (Low) relevance subsample is significantly weaker, absent or (more) negative than in the Low (High) relevance subsample. The edges in panel a show whether the relation is positive or negative; c) Node statistic Strength of the two networks. See Figure 1b in the main text interpretation guidelines.

Table A6.1 – Edge weights corresponding to the networks presented in Figure A1a.

|  | **Age** | **Bio_AmbiDef** | **Bio_BelEnvir** | **Bio_BelHealLong** | **Bio_BelSafe** | **Bio_BelUse** | **Bio_Control** | **Bio_Emo** | **Bio_Norm** | **Bio_Trust** | **Cur_AmbiDef** | **Cur_BelNeg** | **Cur_BelPos** | **Cur_BelRecy** | **Cur_BelUse** | **Cur_Control** | **Cur_EmoNeg** | **Cur_EmoPos** | **Cur_Norm** | **Cur_Think** | **Cur_Trust** | **Exposure** | **Health** | **PrefExt** | **PrefInt** |
| --- | --- | --- | --- | --- | --- | --- | --- | --- | --- | --- | --- | --- | --- | --- | --- | --- | --- | --- | --- | --- | --- | --- | --- | --- | --- |
| **Low relevance** |  |  |  |  |  |  |  |  |  |  |  |  |  |  |  |  |  |  |  |  |  |  |  |  |  |
| Age |  | -.04 |  |  | .00 |  |  |  |  |  |  |  |  |  |  |  |  |  |  |  |  |  |  |  |  |
| Bio_Ambi |  |  | -.12 |  |  |  |  | -.06 | -.06 |  | .05 |  | -.12 |  |  |  |  |  |  |  |  |  |  |  |  |
| Bio_BelEnvir |  |  |  | .09 |  |  |  | .33 | .13 | .05 |  | .02 | .02 |  | -.05 |  |  |  |  |  |  |  |  | .22 |  |
| Bio_BelHealLong | |  |  |  | .15 |  |  | .14 | .04 |  |  | .01 |  |  |  |  |  |  |  | .10 |  |  |  | .00 | .05 |
| Bio_BelSafe |  |  |  |  |  | .12 | .10 | .11 |  |  |  |  |  |  |  |  | .11 |  |  |  |  | -.02 |  | .02 | .09 |
| Bio_BelUse |  |  |  |  |  |  | .07 |  |  |  |  |  |  |  | .37 | .06 |  |  |  |  |  |  |  |  |  |
| Bio_Control |  |  |  |  |  |  |  |  |  | .05 |  |  |  |  |  | .39 | .07 |  |  | .05 |  |  |  |  | .03 |
| Bio_Emo |  |  |  |  |  |  |  |  | .12 | .08 |  | .12 |  |  |  |  | .05 |  |  | .00 |  |  |  | .11 | .16 |
| Bio_Norm |  |  |  |  |  |  |  |  |  | .13 |  |  | .04 |  |  |  |  |  |  |  |  |  |  | .02 | .06 |
| Bio_Trust |  |  |  |  |  |  |  |  |  |  |  |  |  |  |  |  |  |  |  |  | .33 |  | .01 | .11 | .05 |
| Cur_AmbiDef |  |  |  |  |  |  |  |  |  |  |  | .02 |  | -.05 |  |  | .12 | -.01 |  | .03 |  |  |  | .03 |  |
| Cur_BelNeg |  |  |  |  |  |  |  |  |  |  |  |  |  | -.08 | -.05 |  | .31 | -.18 |  | .07 | -.09 |  |  | .16 | .03 |
| Cur_BelPos |  |  |  |  |  |  |  |  |  |  |  |  |  | .07 |  |  | -.23 | .01 | .21 |  | .21 | .07 |  |  |  |
| Cur_BelRecy |  |  |  |  |  |  |  |  |  |  |  |  |  |  | .04 |  | -.07 | .13 | .02 |  | .02 |  |  |  |  |
| Cur_BelUse |  |  |  |  |  |  |  |  |  |  |  |  |  |  |  |  |  | .01 |  |  |  |  |  |  |  |
| Cur_Control |  |  |  |  |  |  |  |  |  |  |  |  |  |  |  |  |  | .03 |  | .11 |  | .04 |  |  |  |
| Cur_EmoNeg |  |  |  |  |  |  |  |  |  |  |  |  |  |  |  |  |  | -.05 |  | .11 | -.17 |  |  |  |  |
| Cur_EmoPos |  |  |  |  |  |  |  |  |  |  |  |  |  |  |  |  |  |  | .04 |  | .18 |  |  |  |  |
| Cur_Norm |  |  |  |  |  |  |  |  |  |  |  |  |  |  |  |  |  |  |  |  | .07 |  |  |  |  |
| Cur_Think |  |  |  |  |  |  |  |  |  |  |  |  |  |  |  |  |  |  |  |  |  | .12 |  |  | .01 |
| Cur_Trust |  |  |  |  |  |  |  |  |  |  |  |  |  |  |  |  |  |  |  |  |  |  | .02 |  |  |
| Exposure |  |  |  |  |  |  |  |  |  |  |  |  |  |  |  |  |  |  |  |  |  |  |  |  |  |
| Health |  |  |  |  |  |  |  |  |  |  |  |  |  |  |  |  |  |  |  |  |  |  |  |  |  |
| PrefExt |  |  |  |  |  |  |  |  |  |  |  |  |  |  |  |  |  |  |  |  |  |  |  |  | .30 |
| PrefInt |  |  |  |  |  |  |  |  |  |  |  |  |  |  |  |  |  |  |  |  |  |  |  |  |  |
| **High relevance** |  |  |  |  |  |  |  |  |  |  |  |  |  |  |  |  |  |  |  |  |  |  |  |  |  |
| Age |  | -.08 |  |  |  |  |  | .01 |  |  |  |  |  |  |  |  | -.05 |  |  |  |  |  | -.04 |  |  |
| Bio_Ambi |  |  | -.22 |  |  | .06 |  | -.10 | -.07 |  | .06 |  | -.03 |  |  | .03 | .09 |  |  |  |  |  |  | -.07 |  |
| Bio_BelEnvir |  |  |  | .13 |  |  |  | .29 | .14 | .07 |  | .09 | .10 |  | -.04 |  |  |  |  |  |  |  |  | .12 |  |
| Bio_BelHealLong | |  |  |  | .28 |  | .03 | .14 | .02 |  |  | .05 |  |  |  |  | .06 |  |  |  |  |  |  | .04 | .04 |
| Bio_BelSafe |  |  |  |  |  | .20 | .09 | .02 |  | .24 |  |  |  | .02 |  | .05 | .01 |  |  | .01 |  |  |  |  | .14 |
| Bio_BelUse |  |  |  |  |  |  | .01 |  |  |  |  |  |  |  | .29 | .08 | .05 |  |  | .07 |  |  |  |  |  |
| Bio_Control |  |  |  |  |  |  |  |  |  | .05 |  |  | -.01 |  |  | .41 | .02 | .01 |  | .10 | .01 | .03 |  |  |  |
| Bio_Emo |  |  |  |  |  |  |  |  | .04 | .08 |  | .03 |  |  | -.03 |  | .03 | -.03 |  |  | -.06 | -.01 |  | .19 | .19 |
| Bio_Norm |  |  |  |  |  |  |  |  |  | .11 |  | .02 |  | .02 |  |  |  |  |  |  |  |  |  | .08 | .06 |
| Bio_Trust |  |  |  |  |  |  |  |  |  |  |  |  |  |  |  |  |  |  |  |  | .36 |  | .01 | .05 | .05 |
| Cur_AmbiDef |  |  |  |  |  |  |  |  |  |  |  | .07 |  | -.07 |  |  | .21 | -.01 | -.01 | .06 |  |  |  | .02 |  |
| Cur_BelNeg |  |  |  |  |  |  |  |  |  |  |  |  |  | -.10 |  |  | .38 | -.11 |  |  | -.09 |  |  | .16 | .02 |
| Cur_BelPos |  |  |  |  |  |  |  |  |  |  |  |  |  | .06 |  |  | -.19 | .13 | .24 |  | .24 | .01 |  |  |  |
| Cur_BelRecy |  |  |  |  |  |  |  |  |  |  |  |  |  |  | .14 | .02 |  | .08 |  |  | .09 | .03 |  |  |  |
| Cur_BelUse |  |  |  |  |  |  |  |  |  |  |  |  |  |  |  | .03 |  |  |  | .04 |  |  |  |  |  |
| Cur_Control |  |  |  |  |  |  |  |  |  |  |  |  |  |  |  |  | .07 |  |  | .14 |  | .03 |  |  |  |
| Cur_EmoNeg |  |  |  |  |  |  |  |  |  |  |  |  |  |  |  |  |  | -.01 |  | .27 | -.12 |  |  | .02 | .03 |
| Cur_EmoPos |  |  |  |  |  |  |  |  |  |  |  |  |  |  |  |  |  |  | .06 |  | .20 | .04 |  |  |  |
| Cur_Norm |  |  |  |  |  |  |  |  |  |  |  |  |  |  |  |  |  |  |  |  | .03 |  |  |  |  |
| Cur_Think |  |  |  |  |  |  |  |  |  |  |  |  |  |  |  |  |  |  |  |  |  | .10 |  |  |  |
| Cur_Trust |  |  |  |  |  |  |  |  |  |  |  |  |  |  |  |  |  |  |  |  |  |  |  |  | -.01 |
| Exposure |  |  |  |  |  |  |  |  |  |  |  |  |  |  |  |  |  |  |  |  |  |  | -.23 |  |  |
| Health |  |  |  |  |  |  |  |  |  |  |  |  |  |  |  |  |  |  |  |  |  |  |  | .02 | .05 |
| PrefExt |  |  |  |  |  |  |  |  |  |  |  |  |  |  |  |  |  |  |  |  |  |  |  |  | .26 |
| PrefInt |  |  |  |  |  |  |  |  |  |  |  |  |  |  |  |  |  |  |  |  |  |  |  |  |  |

**Independent groups gaussian network comparison test**

*p*-value correction: none

Global strength invariance test

Global strength per group: low relevance 8.427427; high relevance 10.20951

Test statistic S: 1.782079

*p*-value 0.05194805

Network invariance test

Test statistic M: 0.2411553

*p*-value 0.002997003

Table A6.2 - Edges that differ significantly between the relevance subsamples with *p*-value < .05. Variables that differed significantly between the relevance subsample at *p* < .01 are reported in bold text.

| **Var1** | **Var2** | ***p*-value** | **Test statistic E** |
| --- | --- | --- | --- |
| Bio_BelHealLong | Bio_Control | .026 | .028 |
| **Bio_BelSafe** | **Bio_Trust** | **.001** | **.241** |
| Cur_BelNeg | Cur_BelUse | .012 | .046 |
| **Bio_BelHealLong** | **Cur_EmoNeg** | **.004** | **.056** |
| Bio_BelSafe | Cur_EmoNeg | .030 | .106 |
| Cur_BelRecy | Cur_EmoNeg | .047 | .073 |
| Cur_BelPos | Cur_EmoPos | .021 | .127 |
| Bio_BelUse | Cur_Think | .018 | .069 |
| **Cur_BelUse** | **Cur_Think** | **.002** | **.038** |
| **Cur_EmoNeg** | **Cur_Think** | **.001** | **.169** |
| Bio_Control | Cur_Trust | .017 | .009 |
| **Bio_Control** | **Exposure** | **.005** | **.033** |
| **Cur_BelRecy** | **Exposure** | **.001** | **.029** |
| **Exposure** | **Health** | **.002** | **.229** |
| Bio_Control | PrefInt | .043 | .032 |

Table A6.3 – Node strength values of the total sample. Variables that differed significantly between the relevance subsample at *p* < .05 are reported in bold text.

| Variables | Low | High | *p*-value  if *p* < .05 |
| --- | --- | --- | --- |
| Age | 0.05 | 0.18 |  |
| **Bio_AmbiDef** | **0.45** | **0.83** | **.020** |
| Bio_BelEnvir | 1.03 | 1.18 |  |
| Bio_BelHealLong | 0.58 | 0.78 |  |
| **Bio_BelSafe** | **0.72** | **1.05** | **.011** |
| Bio_BelUse | 0.62 | 0.76 |  |
| Bio_Control | 0.76 | 0.78 |  |
| Bio_Emo | 1.27 | 1.24 |  |
| Bio_Norm | 0.58 | 0.55 |  |
| Bio_Trust | 0.80 | 1.02 |  |
| Cur_AmbiDef | 0.30 | 0.51 |  |
| Cur_BelNeg | 1.14 | 1.11 |  |
| Cur_BelPos | 0.98 | 1.02 |  |
| Cur_BelRecy | 0.47 | 0.63 |  |
| Cur_BelUse | 0.52 | 0.56 |  |
| Cur_Control | 0.62 | 0.87 |  |
| **Cur_EmoNeg** | **1.29** | **1.62** | **.015** |
| Cur_EmoPos | 0.63 | 0.68 |  |
| Cur_Norm | 0.33 | 0.33 |  |
| Cur_Think | 0.61 | 0.79 |  |
| Cur_Trust | 1.07 | 1.21 |  |
| Exposure | 0.26 | 0.47 |  |
| **Health** | **0.02** | **0.35** | **.010** |
| PrefExt | 0.97 | 1.05 |  |
| PrefInt | 0.78 | 0.87 |  |

**References**

1. Thompson MM, Zanna MP, Griffin DW. Let’s not be indifferent about (attitudinal) ambivalence. Attitude strength: Antecedents and consequences. 1995;4:361-86.

2. Kaplan KJ. On the ambivalence-indifference problem in attitude theory and measurement: A suggested modification of the semantic differential technique. Psychological Bulletin. 1972;77:361-72.

3. Epskamp S, Borsboom D, Fried EI. Estimating psychological networks and their accuracy: A tutorial paper. Behavior Research Methods. 2018;50(1):195-212.
